# Sclerostin inhibits Wnt signaling through tandem interaction with two LRP6 ectodomains

**DOI:** 10.1038/s41467-020-19155-4

**Published:** 2020-10-23

**Authors:** Jinuk Kim, Wonhee Han, Taeyong Park, Eun Jin Kim, Injin Bang, Hyun Sik Lee, Yejin Jeong, Kyeonghwan Roh, Jeesoo Kim, Jong-Seo Kim, Chanhee Kang, Chaok Seok, Jin-Kwan Han, Hee-Jung Choi

**Affiliations:** 1 Department of Biological Sciences, Seoul National University, Seoul, 08826 Republic of Korea; 2 Department of Life Sciences, Pohang University of Science and Technology, Pohang, Gyeongbuk 37673 Republic of Korea; 3 Department of Chemistry, Seoul National University, Seoul, 08826 Republic of Korea; 4 School of Pharmacy, Sungkyunkwan University, Suwon, 16419 Republic of Korea; 5 Center for RNA Research, Institute for Basic Science, Seoul, 08826 Republic of Korea; 6 Present Address: Plumbline Life Sciences, Inc., Seoul, 06552 Republic of Korea; 7 Present Address: Department of Physiology and Cellular Biophysics, Columbia University Irving Medical Center, New York, NY 10032 USA

**Keywords:** Intracellular signalling peptides and proteins, X-ray crystallography

## Abstract

Low-density lipoprotein receptor-related protein 6 (LRP6) is a coreceptor of the β-catenin-dependent Wnt signaling pathway. The LRP6 ectodomain binds Wnt proteins, as well as Wnt inhibitors such as sclerostin (SOST), which negatively regulates Wnt signaling in osteocytes. Although LRP6 ectodomain 1 (E1) is known to interact with SOST, several unresolved questions remain, such as the reason why SOST binds to LRP6 E1E2 with higher affinity than to the E1 domain alone. Here, we present the crystal structure of the LRP6 E1E2–SOST complex with two interaction sites in tandem. The unexpected additional binding site was identified between the C-terminus of SOST and the LRP6 E2 domain. This interaction was confirmed by in vitro binding and cell-based signaling assays. Its functional significance was further demonstrated in vivo using *Xenopus laevis* embryos. Our results provide insights into the inhibitory mechanism of SOST on Wnt signaling.

## Abstract (web summary)

The low-density lipoprotein receptor-related protein 6 (LRP6) is a co-receptor of the β-catenin-dependent Wnt signaling pathway and interacts with the Wnt inhibitor sclerostin (SOST). Here the authors present the crystal structure of SOST in complex with the LRP6 E1E2 ectodomain construct, which reveals that the SOST C-terminus binds to the LRP6 E2 domain, and further validate this binding site with in vitro and in vivo experiments.

## Introduction

**Fig. 1:**
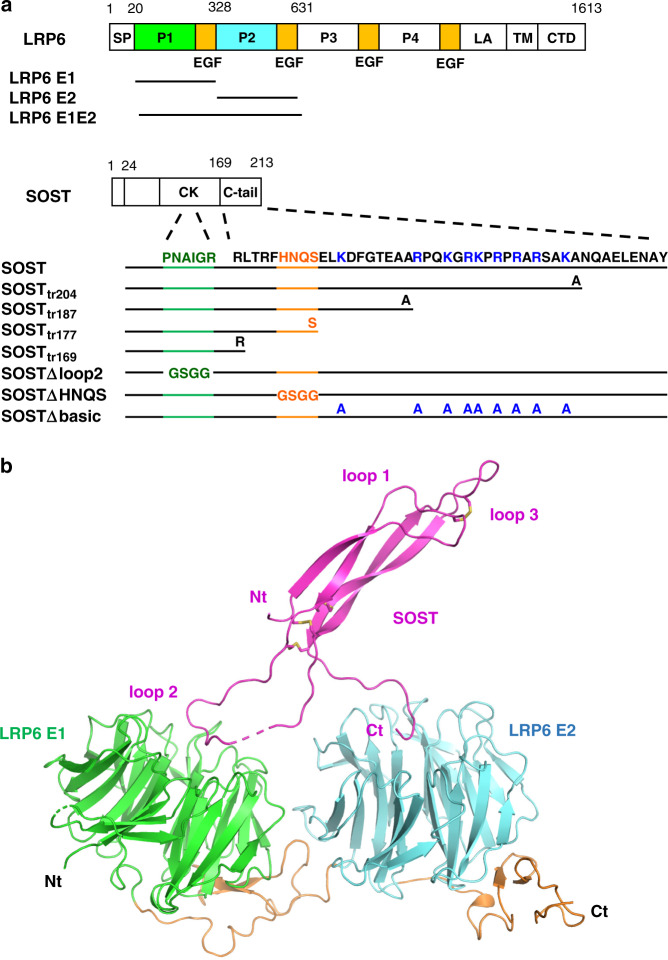
Construct design and the crystal structure of the LRP6 E1E2–SOST_tr177_ complex. **a** Schematic representations of the LRP6 and SOST constructs. SP signal peptide, P β-propeller domain, EGF epidermal growth factor-like domain, LA LDLR type-A repeats, TM transmembrane domain, CTD C-terminal domain, CK cystine-knot domain. The amino acid residues interacting with LRP6 E1 in the SOST-loop 2 region are shown in dark green. The SOST C-terminal tail sequences including HNQS (orange) and the basic-residue clusters (blue) are also shown. Construct information is described in detail in Supplementary Table 1. **b** The crystal structure of the LRP6 E1E2–SOST_tr177_ complex is represented with a ribbon diagram. For simplicity, only one complex out of two in an asymmetric unit is shown (Supplementary Fig. 4). SOST is colored in magenta, and disulfide bonds are represented as yellow sticks. SOST residues 122–127 are not resolved in this structure and are represented as a dashed line. The LRP6 E1 β-propeller domain is colored in green, and the E2 β-propeller is shown in cyan. The EGF domains are shown in orange.

Low-density lipoprotein (LDL) receptor-related protein 5/6 (LRP5/6) is an essential co-receptor of the canonical Wnt signaling pathway. As a member of the LDL receptor family, LRP6 is a single-pass transmembrane protein with a large extracellular domain (ECD) consisting of four repeating units (E1, E2, E3, and E4), each of which has a YWTD β-propeller domain flanked by an epidermal growth factor-like domain (Fig. 1a)^1^. The ECD of LRP5/6 is an important regulatory site for Wnt signaling. It provides at least two independent binding sites for Wnt ligands, as supported by the observed simultaneous binding of Wnt3a and Wnt9b to the LRP6 ECD^2^. Although structural information regarding the Wnt–LRP6 complex is yet to be determined, biochemical studies have shown that Wnt3a and Wnt9b interact with LRP6 E3E4 and E1E2, respectively, with *K*_D_ values in the range of 10–100 nM^2^. In addition to Wnts, various Wnt signaling inhibitors, including Dickkopf-1 (DKK1), sclerostin (SOST), and sclerostin domain-containing protein 1 (SOSTDC1, also called as WISE), are known to bind to LRP6 ECD^3–5^.

First identified in patients suffering from sclerosteosis, SOST is a secreted glycoprotein that is predominantly expressed in osteocytes^6,7^. SOST inhibits new bone formation and growth by binding to LRP5/6, thereby inhibiting canonical Wnt signaling in osteoblasts^3,8^. Sclerosteosis, caused by defects in the *SOST* gene, is characterized by abnormally high bone mass^6^. This high bone density phenotype was also observed in SOST-null mice^9^. This phenotypic characterization makes SOST a compelling therapeutic target for osteoporosis^10^.

Structural analysis of SOST using nuclear magnetic resonance (NMR) spectroscopy showed that it forms a core cystine-knot structure with three loops, flanked by highly flexible N- and C-terminal domains^11^. Unlike most cystine-knot family members, which form homodimers or heterodimers (such as BMP-7 and noggin)^12^, SOST may function as a monomer. The LRP6-interaction site on SOST was mapped to the NXI motif in loop 2 using NMR spectroscopy, which was later confirmed by the crystal structure of SOST loop 2 peptide-bound LRP6 E1^13^. Although clear evidence indicates that the SOST loop 2 region serves as the primary interaction site with LRP6 E1, previous data raised the possibility that an additional interaction site exists between SOST and LRP6. For example, the IC_50_ of the SOST loop 2 peptide, measured by Förster resonance energy transfer and enzyme-linked immunosorbent assay (ELISA) analyses, is remarkably large (14–21 μM)^13,14^, considering that the *K*_D_ value of full-length SOST for LRP6 E1E2 was determined as 6.8 nM using biolayer interferometry^13^. In addition, LRP6 E1 bound SOST with an approximately 10-fold lower affinity than LRP6 E1E2^13^.

To determine whether any region of SOST other than loop 2 participates in the interaction with LRP6, we performed structural, functional, and biochemical analyses of SOST. The crystal structure of a complex between LRP6 E1E2 and SOST_tr177_ (a C-terminal 37-amino acid truncation mutant of SOST) suggested that the C-terminal region of SOST might contribute to LRP6 binding. The functional significance of this additional binding site was confirmed through TopFlash assays, binding-affinity measurements, and in vivo experiments using *Xenopus laevis* embryos. Collectively, our data show that loop 2 and the C-terminal tail (C-tail) of SOST participate in LRP6 binding, bringing a new perspective to the inhibitory mechanism of SOST in the LRP6-mediated Wnt signaling.

## Results

### Crystal structure of LRP6–SOST_tr177_ complex

Based on the published NMR structure of SOST, we designed SOST_tr177_ (Supplementary Table 1) for crystallization purpose and co-purified it with two N-terminal subdomains of LRP6 E1E2 (Fig. 1a). The crystal structure of the LRP6 E1E2–SOST_tr177_ complex was solved at 3.8 Å resolution by the molecular replacement (MR) method using the known LRP6 E1E2 structure (PDB ID 3S94) and the core cystine-knot NMR structure of SOST (PDB ID 2K8P) with the loop 2 region removed as search models. Despite the low-resolution X-ray data, calculated electron density maps were of sufficient quality to build and refine SOST and nearly all of the LRP6 E1 and E2 domains. The mFo-DFc difference electron density map calculated after rigid body fitting of the MR solution revealed clear density for SOST NXI motif at the top surface of LRP6 E1 (Supplementary Fig. 1). In addition, we observed an unidentified electron density at the center of the LRP6 E2 propeller in two independent copies of LRP6 molecules in an asymmetric unit of the crystal lattice (Supplementary Fig. 1). A possibility that a second SOST molecule bound LRP6 E2 was excluded by the fact that 1:1 heterodimer formation was observed for the LRP6 E1E2–SOST_tr177_ complex using multi-angle light scattering (MALS) analysis (Supplementary Fig. 2). Moreover, continuous electron density from the C-terminus of the SOST cystine-knot core to the top of LRP6 E2 was observed (Supplementary Fig. 3). The shortened C-tail of our SOST_tr177_ construct (169-RLTRFHNQS-177) facilitated model building, as we observed a clear side-chain density for H174 at the center of LRP6 E2, where H174 stably interacts with polar residues of LRP6 E2. Thus, the last four residues, HNQS, were successfully modeled into the electron density on the top of LRP6 E2 (Fig. 1b). The SOST polypeptide 78–121 and 129–177 in chain C and 78–121 and 131–177 in chain D were included in the final model. In both SOST molecules, we did not observe direct crystal contact between the loop 2 region or the C-tail region and neighboring molecules (Supplementary Fig. 4).

To support our crystal structure demonstrating the interaction between SOST HNQS and LRP6 E2, mass spectrometric (MS) analysis was performed following reaction with the crosslinker, bis(sulfosuccinimidyl) suberate (BS3). Based on the crystal structure of the LRP6 E1E2–SOST_tr177_ complex, we introduced the H404K and N175K mutations into LRP6 E1E2 and SOST_tr177_, respectively (Supplementary Fig. 5), and purified the LRP6 E1E2–SOST_tr177_ complex with both mutations, which was crosslinked with BS3 crosslinker. By tandem MS, intermolecular crosslinking between the SOST fragment (H*K*QS) and the LRP6 fragment (397-VVTAQIA*K*PDGIAVDW-412) was clearly identified (Supplementary Fig. 5), suggesting that the SOST HNQS is located close to H404 of LRP6 E2 in the LRP6 E1E2–SOST_tr177_ complex.

Comparing our LRP6–SOST complex structure with that of the free SOST structure (PDB ID 2K8P) shows that, while the N-terminal region of SOST remained highly flexible as it was unresolved in our structure (residues 24–77), loop 2 and the HNQS region were adjusted to accommodate LRP6 binding (Supplementary Fig. 6). Since our SOST construct ends immediately after the HNQS sequence, our structure did not enable us to determine whether the remaining C-terminus would make additional contacts with LRP6 or remain free. The possibility of further interaction mediated by the SOST C-terminus is discussed below. However, the current structure clearly indicates that SOST interacts with both LRP6 E1 and E2 through loop 2 and C-tail, respectively.

### Comparing two SOST-binding sites on LRP6

**Fig. 2:**
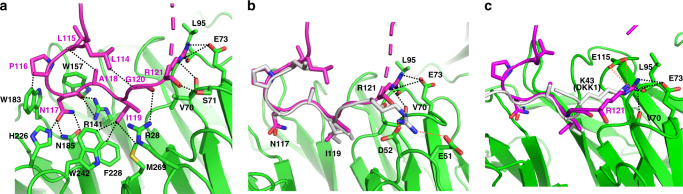
Canonical binding site of SOST loop 2 in LRP6 E1. **a** Key interactions of the SOST loop 2 region with LRP6 E1 (within 4 Å) are shown with dotted lines. Interacting residues are presented as sticks and are labeled black for LRP6 or magenta for SOST. The missing region of SOST (residues 122–127) in this structure is represented with a dashed line. **b** Comparison of the SOST loop 2 binding site in our structure with that of the LRP6 E1–SOST peptide complex structure (PDB ID: 3SOV) is presented. The SOST “LPNAIGR” peptide is shown in light gray, and LRP6 E1 from PDB 3SOV is omitted for clarity. The interactions of the NXI motif were found to be conserved in both structures, although the R121-mediated interactions differed. The interactions of R121 in the LRP6 E1-peptide structure are represented with orange dotted lines. **c** Structural comparison with the LRP6 E1–DKK1 peptide (NSNAIKN) complex (PDB ID: 3SOQ) is shown. The DKK1 peptide is colored in light gray. The interactions of K43 in the LRP6 E1–DKK1 peptide structure are represented in orange dotted lines.

The previously published structure of LRP6 E1 in complex with the SOST loop 2 peptide established SOST N117 and I119 in the NXI motif as being critical for LRP6 E1 binding. This was confirmed by a 10-fold lower affinity of the N117A and I119E SOST mutants for LRP6^13^. While we observed similar interactions between the NXI motif and the LRP6 E1-binding surface here, the intact loop 2 of SOST exhibited two interesting structural features that were not observed in the peptide-complex structure (PDB ID 3SOV). Firstly, SOST L114 and L115 formed van der Waals interactions with SOST A118 and G120, respectively, thereby stabilizing the loop 2 conformation (Fig. 2a), which may contribute to the overall stability of the LRP6–SOST complex. Another difference was in the interaction of SOST R121. In our structure, R121 made close contacts with the E73 side chain and the backbone carbonyls of V70 and L95 in LRP6 E1 (Fig. 2b). However, in the peptide-complex structure, R121 instead interacted with E51 and D52 of LRP6 E1 with its carboxyl group pointing toward the LRP6 core. This configuration is not possible for R121 in the intact SOST protein, as it does not allow space for the remaining polypeptide chain. When compared to the structure of the NXI motif-containing DKK1 peptide bound to LRP6 E1, we noticed that R121 in our SOST structure aligned well with K43 of DKK1, even though K43 followed the NXI motif without an intervening Gly residue (NXIK), in contrast to SOST (NXIGR). DKK1 K43 interacts with the same carbonyl of L95, but with the E115 side chain of LRP6 (Fig. 2c). Thus, the side chain orientation of this positively charged residue following the NXI motif (toward the acidic region on LRP6 E1) appears to be conserved in DKK1 and SOST, regardless of whether an extra Gly residue is present before the basic residue.

**Fig. 3:**
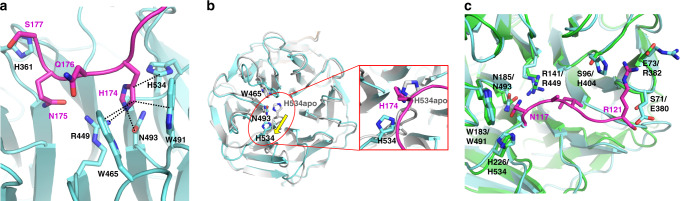
Interaction of SOST C-tail with LRP6 E2. **a** The HNQS motif of the SOST C-terminus fits into the binding pocket of the LRP6 E2 β-propeller domain. Interactions of SOST H174 with LRP6 residues are shown with black dotted lines. **b** The ligand-free state of LRP6 E2 (PDB ID: 3S94; light gray) was aligned to LRP6 E2 (cyan) in our SOST-bound structure. For clarity, SOST was omitted from the overall alignment of  LRP6 E2, on the left. Structural changes near W465 and H534 in LRP6 E2 upon SOST binding are shown. H534 of LRP6 moved from the concave center of the β-propeller domain by binding to SOST H174 (colored in magenta in an enlarged box on the right). **c** The structure of HNQS-bound LRP6 E2 (cyan) was aligned with the structure of loop 2-bound LRP6 E1 (green). The HNQS region is not shown for clarity. Loop 2-interacting residues in LRP6 E1 and corresponding residues in LRP6 E2 are represented as sticks and E2 residues are labeled after the E1 residues.

At the LRP6 E2 binding site, the side chain of H174 in the HNQS was modeled into the central cavity of the LRP6 E2 β-propeller by fitting it to the electron density map (Supplementary Fig. 3). H174 of SOST made close contacts with R449, W465, W491, N493, and H534 of LRP6 (Fig. 3a). Unexpectedly, in contrast to LRP6 E1, LRP6 E2 showed conformational changes upon SOST binding in that residues at the center of the LRP6 E2 propeller were rearranged to accommodate the insertion of SOST H174 (Fig. 3b). Unlike the LRP6 E1 and E3 propellers, the top surface of the LRP6 E2 propeller has a loop that bulges out between blades 5 and 6 in the ligand-free state^1^. Structural analysis of the LRP6 E1E2–SOST_tr177_ complex showed that SOST H174 replaced the H534 residue of LRP6, located on this protruding loop that interacts with W465 and N493 in the ligand-free structure of LRP6 E2 (PDB ID 3S94). This loop rearrangement appears to be pivotal for allowing ligand binding to the top surface of LRP6 E2.

The ligand-binding pocket of the SOST C-tail-bound LRP6 E2 (consisting of W491, N493, R449, and H534) appears remarkably similar to that of the SOST loop 2-bound LRP6 E1 (Fig. 3c). However, while the NAI residues of loop 2 fit into the LRP6 E2 cavity, the potential interactions around SOST R121 are unfavorable in LRP6 E2, suggesting that R121 contributes to selective binding of SOST loop 2 to LRP6 E1. R121 can form ionic interaction with E73 in LRP6 E1, but the corresponding residue in LRP6 E2 is R382. The charge repulsion between the two Arg residues would be expected to prevent SOST loop 2 binding to LRP6 E2. In addition, the R121 backbone and side chain would clash with E380 and H404 in the LRP6 E2, if the two were to bind (Fig. 3c).

We noted that N175 in the HNQS sequence was predicted to be a glycosylation site. In our SOST_tr177_ construct, N175 was not glycosylated. However, purified wild-type (WT) SOST revealed a doublet band following sodium dodecyl sulfate-polyacrylamide gel electrophoresis (SDS-PAGE; Supplementary Fig. 7). This doublet was converted to a single band by PNGase F treatment to induce deglycosylation. These results indicate that, in vivo, N175 in WT SOST is likely glycosylated. However, because the N175 side chain points toward the solvent region, glycosylation at this site is unlikely to interfere with the LRP6 interaction (Supplementary Fig. 8).

This is the first report showing a direct ligand interaction of LRP6 E2. While the ligand-bound and ligand-free structures of LRP6 E1 were similar, structural changes in LRP6 E2 were observed upon ligand binding. To verify the functional impact of the interaction of the SOST C-tail with LRP6 E2, we performed in vitro-binding experiments and cell-based assays.

### Evaluation of the role of SOST C-tail in LRP6 binding

Based on our structure, it was unclear whether the C-terminus following HNQS promotes the interaction with LRP6. Attempts at crystallizing LRP6 in complex with full-length SOST were unsuccessful. However, the sequence alignment of SOST and its homolog WISE revealed that, unlike the N-terminal region, the C-terminal region contains conserved residues, implying its functional importance (Supplementary Fig. 9). The conserved sequence encompasses HNQ(E)S and also includes a previously unnoted cluster of basic residues. A series of SOST truncation mutants, with terminal residues of 169, 177, and 204, were generated to evaluate the importance of the HNQS motif and the basic residues (Fig. 1a). SOST_tr204_ contained both the HNQS motif and a basic-residue cluster, excluding only the last nine non-conserved amino acids of the SOST C-terminus. In contrast, SOST_tr169_ contained neither the HNQS region nor the basic-residue cluster. Finally, SOST_tr177_ contained only the HNQS motif, but not the basic cluster. The binding affinity of each SOST mutant to LRP6 E1E2 was measured using microscale thermophoresis (MST) and compared with that of WT SOST. WT SOST showed the strongest binding to LRP6 E1E2, with a *K*_D_ of 170 nM (Supplementary Fig. 10 and Supplementary Table 2). Previously, the dissociation constant of SOST for LRP6 E1E2 was determined to be 6.8 nM using biolayer interferometry^13^. This discrepancy may result from the different methods used to determine the *K*_D_ values or the use of the N-terminal SUMO tag (Smo-SOST) to improve the solubility of SOST in MST measurements. However, we confirmed that SUMO itself did not bind LRP6 and that a different N-terminal tag, i.e., maltose-binding protein (MBP), did not affect the affinity for LRP6 (Supplementary Fig. 10). As the N-terminal 54 amino acids of SOST were not resolved in our crystal structure, we hypothesized that the N-terminal region of SOST would not be involved in LRP6 binding. To further verify the *K*_D_ value, we performed size-exclusion chromatography analysis to determine an approximate *K*_D_ value, using unlabeled and untagged proteins. This value (~50 nM) was slightly lower than obtained in our MST experiment (Supplementary Fig. 11).

**Fig. 4:**
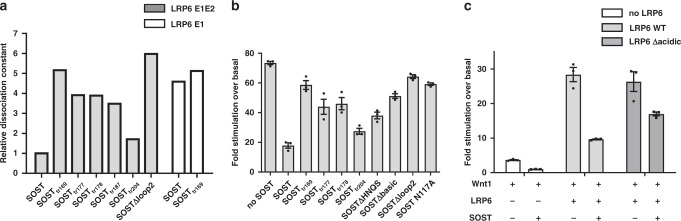
Effects of the SOST C-tail on LRP6 binding and Wnt1-signaling inhibition. **a** The affinities of various SOST mutants for LRP6 E1E2 or LRP6 E1 were measured by MST, and the relative *K*_D_ values from those measurements are shown. The *K*_D_ value of SOST WT for LRP6 E1E2 was used as a reference. The experimentally determined *K*_D_ values are provided in Supplementary Table 2. **b** The inhibitory effects of SOST mutants on Wnt1 signaling were detected by TopFlash assays. Each luciferase signal observed after co-transfecting plasmids encoding Wnt1 and each SOST mutant was normalized to that found with the empty-vector control. **c** Differences in the inhibitory activities of SOST with WT LRP6 or the LRP6Δacidic mutant in LRP6-knockout HEK293T cells are shown in the bar graph. Data from three independent experiments (*n* = 3) were analyzed and expressed as mean ± SEM in **b** and **c**.

Despite this variation, our results clearly provide reliable information regarding the relative affinities of the SOST mutants and WT SOST (Fig. 4a). As expected, deleting the NXI motif reduced the affinity of SOST for LRP6. This SOST∆loop2 mutant, in which the loop 2 residues were replaced with GSGG (Supplementary Table 1), showed the lowest affinity for LRP6 E1E2 among all tested SOST mutants (Supplementary Table 2 and Supplementary Fig. 10). Whereas WT SOST showed ~ 5-fold lower affinity for LRP6 E1 than LRP6 E1E2, SOST_tr169_ was shown to bind LRP6 E1 and E1E2 with similar low affinities. These results demonstrate that the SOST C-terminus is required for full SOST affinity for LRP6 E1E2 by interacting with LRP6 E2. The contribution of HNQS to the interaction with LRP6 E1E2 was shown by the stronger affinity of SOST_tr177_ to LRP6 E1E2 than that of SOST_tr169_ to LRP6 E1E2. The similar binding affinity of SOSTtr_177_ and SOSTtr_178_ for LRP6 E1E2 confirmed that N175 glycosylation did not interfere with LRP6 binding. Interestingly, the crystallization construct SOST_tr177_ did not exhibit full affinity, whereas SOST_tr204_ did (Supplementary Table 2). These results suggest that residues 178–204 may further interact with LRP6. Notably, a cluster of basic residues was observed in this region. As LRP6 E1E2 contained negative patches on its surface (Supplementary Fig. 12), we speculated that the positive C-terminus of SOST interacts with an acidic patch on LRP6 E1E2. Computational loop modeling proposed that the SOST C-terminus may interact with an acidic patch on LRP6 E2 (Supplementary Fig. 12).

The previously reported *K*_D_ of SOST loop 2 peptide for LRP6 E1 was 9 μM, as measured by isothermal calorimetry^14^. Using MST, we measured the *K*_D_ values of the circularized SOST loop 2 peptide for LRP6 E1 and E1E2 as 5.2 and 5.5 μM, respectively (Supplementary Fig. 13). We then attempted to measure the *K*_D_ of the SOST C-terminal peptide for LRP6 E2, but were unsuccessful due to the aggregation of labeled LRP6 E2 during MST experiment. However, we could infer the effect of C-terminal binding to LRP6 E2 by comparing the *K*_D_ values of the SOST C-terminal peptide with LRP6 E1E2 and E1. A C-terminal peptide containing residues R170–Y213 bound to LRP6 E1E2 with a *K*_D_ of 5.8 μM (Supplementary Fig. 13 and Supplementary Table 2); however, it did not bind LRP6 E1, suggesting that the SOST C-terminal region specifically binds LRP6 E2.

Collectively, our systematic analysis of binding affinity between SOST and LRP6, together with computational modeling, clearly shows that the SOST C-terminal region contributes to the high-affinity SOST–LRP6 interaction.

### Functional implications of the SOST C-tail in Wnt1-signaling inhibition

Next, we determined whether the SOST C-terminus could influence the inhibitory function of SOST on the Wnt1 signaling pathway. Cells were co-transfected with plasmids encoding each SOST mutant and Wnt1, and the inhibition of Wnt1-induced canonical signaling was measured in TopFlash assays. Expression levels of SOST mutants and WT SOST were assessed by ELISA and western blotting (Supplementary Fig. 14). Wnt1 signaling sharply decreased after co-transfection with WT SOST (Fig. 4b). In contrast, the SOST∆loop2 and N117A mutants showed impaired inhibition of Wnt1 signaling, consistent with previously published result^15^.

Consistent with our in vitro-binding data, SOST_tr169_ showed reduced inhibition of Wnt1 signaling compared to WT SOST, even though it contained the intact loop 2, suggesting that the C-tail is important for SOST function. Next, we separately evaluated the HNQS motif and the basic cluster region in C-tail. Two constructs lacking a basic region, SOST_tr187_ and SOST_tr177_, showed less inhibitory activity than WT SOST, but more activity than SOST_tr169_ with the entire C-tail removed (Fig. 4b). In addition, we generated a SOST∆HNQS mutant in which 172–178 (RFHNQSE) were substituted with a GSGG linker (Supplementary Table 1). This mutant showed ~50% inhibition of Wnt1 signaling (Fig. 4b), demonstrating that the C-terminal HNQS motif contributed to SOST function, but was not sufficient for full activity. Next, the H174A mutation was introduced into SOST_tr177_ (SOST_tr177_H174A). Unlike the SOST_tr177_F173A mutant, which showed the same cellular activity as SOST_tr177_, the SOST_tr177_H174A mutant exhibited weaker activity than SOST_tr177_ and similar activity to SOST_tr169_, in support of the importance of H174 as seen from our crystal structure (Supplementary Fig. 15). In addition to C-tail truncation mutants, a SOST∆basic mutant, in which all basic residues in the C-terminus were replaced with Ala residues (Fig. 1a), was used to investigate the functional importance of the C-terminal basic cluster. This mutant inhibited Wnt1 signaling similarly to that of the C-terminal truncation mutant SOST_tr187_, revealing the importance of the conserved basic residues for inhibition by SOST.

Our computational model suggests that the acidic region on LRP6 E2 drove interactions with the SOST C-terminal basic cluster (Supplementary Fig. 12). To verify this, we designed an LRP6 mutant (LRP6Δacidic), where the acidic residues, E529, D530, E564, E566, and D570 of LRP6 E2, were replaced with Lys, and evaluated its function in TopFlash assays with an LRP6-knockout cell line (Supplementary Fig. 16). Transfecting the LRP6Δacidic mutant into LRP6-knockout cells reduced the inhibitory effect of SOST on Wnt1 signaling, compared to WT LRP6 (Fig. 4c), suggesting that these acidic residues on LRP6 E2 participated in SOST binding. The mutated acidic residues in LRP6 E2, except for E564, are conserved in LRP5 (Supplementary Fig. 17).

WISE, a SOST homolog, has been reported to inhibit Wnt1 and Wnt8 signaling by binding to LRP6^5,16^. As mentioned above, sequence analysis showed that the HNQ(E)S motif and the basic cluster in the C-tail, and loop2 are conserved in WISE and SOST. To test whether the WISE C-tail affects WISE-mediated Wnt1-signaling inhibition, TopFlash assays were performed using WT and three WISE mutants. WISE∆loop2, WISE_tr175_, and WISE_tr167_ constructs correspond to SOST∆loop2, SOSTtr_177_, and SOST_tr169_, respectively (Supplementary Figs. 9 and 14). Although WT WISE showed weaker activity than WT SOST, loop2 deletion or C-tail truncation in WISE resulted in 60–75% lower activity than WT WISE (Supplementary Fig. 18), suggesting the functional importance of the WISE C-tail for WISE-dependent Wnt1-signaling inhibition.

Collectively, our cell-based functional assays demonstrate that both the HNQS region and a positively charged cluster are important for the full inhibitory effect of SOST on Wnt1 signaling and that they exhibit additive effects on SOST activity.

### Functional analysis of SOST C-terminus in *X. laevis* embryos

**Fig. 5:**
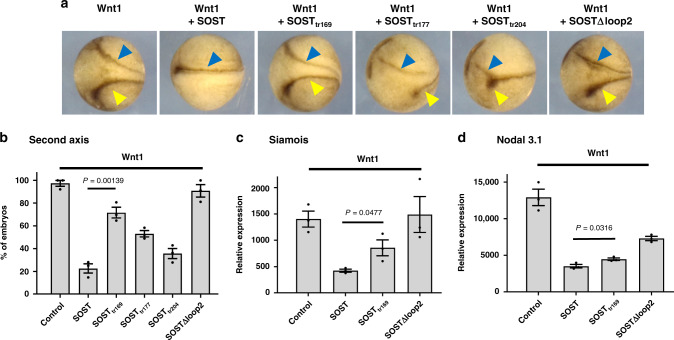
Assessing the inhibitory effect of SOST on Wnt signaling in *Xenopus* embryos. **a** The transverse dark-brown line (blue arrow) indicates the normal position of the axis. The yellow arrow indicates the position of the second ectopic axis. Wnt1 induced formation of the second ectopic axis, and successfully inhibiting Wnt resulted in the formation of a single axis. Embryos at the four-cell stage were co-injected in a ventro-vegetal blastomere with 30 pg *Wnt1* mRNA (*n* = 104), 25 pg WT *SOST* mRNA (*n* = 106), 25 pg *SOST*_*tr169*_ mRNA (*n* = 98), 25 pg *SOST*_*tr177*_ mRNA (*n* = 119), 25 pg *SOST*_*tr204*_ mRNA (*n* = 104), and 25 pg *SOST∆loop2* mRNA (*n* = 96) (*n* number of embryos). **b** The percentage of embryos with a second axis. The error bars represent the standard error of the mean (SEM) from three independent experiments (*n* = 3) and *p* value by two-tailed *t*-test is indicated. The number of injected embryos is described in **a**. **c**, **d** Real-time qPCR analyses of the expression of genes directly targeted by Wnt1, namely *siamois* (**c**) and *nodal 3.1* (**d**) from animal cap explants isolated at stage 8 and grown to stage 11. The error bars represent SEMs from three independent experiments (*n* = 3) and *p* values by two-tailed *t*-test are indicated.

We also investigated the biological importance of the SOCT C-tail in vivo using *X. laevis* embryos^17^. We first confirmed that human LRP6 residues that interact with SOST are conserved in *X. laevis* LRP6 (Supplementary Fig. 17). To validate the functional significance of SOST C-terminal binding, we prepared five *SOST* mRNA constructs (WT, SOST_tr169_, SOST_tr177_, SOST_tr204_, and SOST∆loop2). Injecting *Wnt1* mRNA into a single ventro-vegetal blastomere at the four-cell stage induced ectopic neural tube structures. Co-injecting WT *SOST* mRNA and *Wnt1* mRNA blocked this effect in >60% of the injected embryos (Fig. 5a, b). In contrast, co-transfecting an equal amount of *SOST*_*tr169*_ mRNA was much less effective than WT *SOST* mRNA in inhibiting ectopic-axis formation and was comparable to the inhibition found with *SOST∆loop2* mRNA (Fig. 5a, b). As the C-terminal tail length increased, greater inhibition of ectopic-axis formation occurred (Fig. 5b), consistent with our cell-based results. However, we noticed that injecting increasing amounts of *SOST*_*tr169*_ mRNA restored Wnt1 inhibition, whereas treatment with increasing amounts of *SOST∆loop2* mRNA did not (Supplementary Fig. 19). These data imply that, during Wnt1-mediated signaling, the SOST loop 2 interaction is more important for blocking Wnt1 binding than the C-tail interaction.

The expression of the Wnt-target genes, namely *siamois* and *nodal 3.1* (also known as *Xnr3*), was analyzed by quantitative polymerase chain reaction (qPCR) analysis, with animal cap explants. SOST_tr169_ reduced the Wnt1-mediated expression of both *siamois* and *nodal 3.1* less efficiently than WT SOST (Fig. 5c, d). Intriguingly, qPCR analysis revealed that SOST∆loop2 completely lost the ability to inhibit Wnt1-target gene expression (Fig. 5c, d), supporting a model wherein SOST loop 2 primarily inhibits the Wnt1-signaling pathway. The major difference in the expression of *siamois* and *nodal 3.1* between SOST_tr169_ and SOST∆loop2 is puzzling, given the similar phenotypic results from the second-ectopic axis assay. Other regulatory mechanisms may act to nullify the effect on suppressing *nodal 3.1* expression, or other unnoticed phenotypic differences may have occurred. Nevertheless, our in vivo analysis with *X. laevis* clearly demonstrate that two-site interaction of SOST with LRP6 E1 and E2 are important for Wnt antagonism by SOST.

### SOST-dependent inhibition of Wnt signaling by different Wnt subtypes

Next, we examined whether two-site SOST binding could affect the inhibition of different Wnt subtypes. Previously, Wnt9b signaling was blocked by WT SOST, but not by a SOST loop 2 peptide, whereas Wnt1 signaling was inhibited by both^18^. Based on the hypothesis that LRP6 E1 represents the sole key binding site, it was proposed that the steric effect of SOST was chiefly responsible for the difference observed. Based on our discovery of two-site binding, we speculated that the inhibitory effect of SOST may vary for each Wnt subtype, depending on the interaction site and affinity for LRP6.

**Fig. 6:**
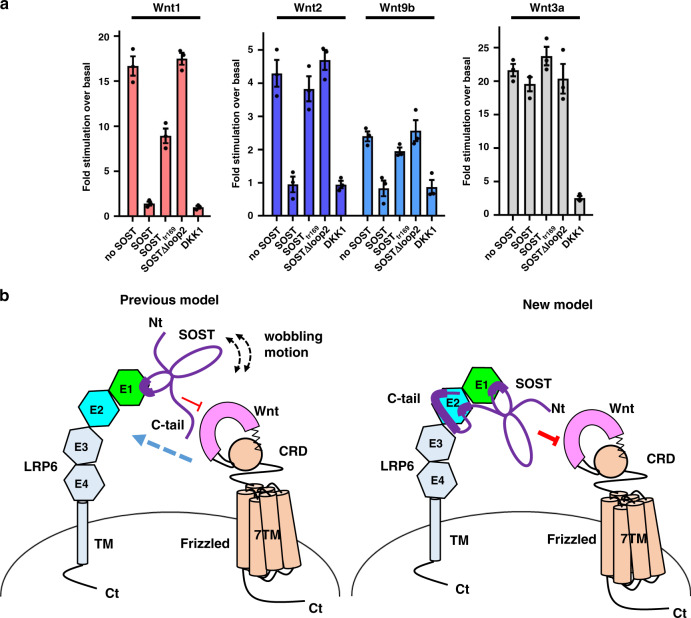
Proposed mechanism whereby SOST inhibits Wnt signaling. The inhibitory effects of WT SOST and SOST mutants on Wnt signaling activated by Wnt1, Wnt2, Wnt9b, and Wnt3a were measured by TopFlash assays. Full-length DKK1 was also used in the activity assays for comparison with SOST. Data from three independent experiments (*n* = 3) were analyzed and expressed as mean ± SEM. **b** A schematic model of the mechanism whereby SOST inhibits the canonical Wnt signaling pathway. In a previous model (left), only the loop 2 region of SOST was suggested to interact with the LRP6 E1 domain, which appears to inefficiently inhibit the binding of various Wnt subtypes. The double-headed arrows indicate the wobbling motion of SOST, owing to flexibility in the loop 2 region. On the other hand, the structural and functional data presented here suggest that the C-terminal HNQS region of SOST binds to LRP6 E2, and that the basic cluster region in the far C-terminus of SOST may further strengthen the interaction with the E2 domain. Based on the SOST C-tail-mediated interaction with E2, we propose a new binding model for SOST (right). In this model, tandem interaction of SOST with LRP6 E1 and E2 leads to more efficient blockage of Wnt binding.

Using Wnt1, Wnt2, Wnt9b, and Wnt3a, we performed TopFlash assays after co-transfection with WT SOST or SOST_tr169_ constructs. A clear difference in inhibition was observed with Wnt3a versus the other Wnt proteins. That is, SOST and DKK1 inhibited Wnt1, Wnt2, and Wnt9b signaling by similar degrees, whereas SOST does not inhibit Wnt3a signaling as much as DKK1 (Fig. 6a and Supplementary Fig. 20). The SOST_tr169_ mutant inhibited Wnt1 signaling (50%) more than Wnt2 and Wnt9b signaling (10%; Fig. 6a), suggesting that efficient Wnt2 and Wnt9b inhibition requires the SOST C-tail. Intriguingly, SOST∆loop2 showed complete abolishment of the inhibitory effect on Wnt1, Wnt2, and Wnt9b signaling. This may reflect the lower affinity of SOST∆loop2 for LRP6. Notably, partial Wnt1-signaling inhibition was achieved with SOST_tr169_, but not by SOST∆loop2, despite their similar affinities for LRP6 E1E2 (*K*_D_ values of 0.9 μM versus 1.0 μM). These results indicate that the Wnt1 binding site on LRP6 may overlap with that of SOST loop 2.

WT SOST exhibited different inhibitory effects on each Wnt subtype. Strong inhibition of Wnt1 signaling occurred following co-transfection with SOST, whereas relatively lower inhibition of Wnt2 and Wnt9b signaling occurred (Fig. 6a). These data may reflect differences in the affinity of each Wnt subtype for LRP6. Binding of Wnt1 to LRP6 appears to be weaker than that of Wnt2 or Wnt9b; thus, Wnt1 is easily released from LRP6 by SOST. Therefore, SOST can provide a diverse spectrum of Wnt inhibition for various Wnts depending on their LRP6-binding affinities and LRP6-binding sites.

## Discussion

SOST is abundantly expressed in osteocytes and functions as an important regulator of bone homeostasis. Among the 19 human Wnts, Wnt1, 3a, 4, 5a, 5b, 7b, 9b, and 10b have been detected in bone tissues and osteoblasts and found to participate in new bone formation^19^. Wnt2, 4, 5a, 11, and 16 were expressed in bone mesenchymal stem cells and implicated in injury repair via osteogenic differentiation^20^. If the SOST-binding site was restricted to LRP6 E1, only a limited subgroup of Wnts, whose binding sites are on the top surface of the LRP6 E1 β-propeller, would be regulated by SOST (Fig. 6b). Here, we discovered an additional SOST-binding site in LRP6 E2 that increases the binding affinity of SOST for LRP6 and promotes more effective blocking of various Wnts (Fig. 6b), similar to previous observations with DKK1. The concomitant binding of DKK1 to LRP6 E1 and E3 leads to inhibited activity of various Wnts^21^. Moreover, the synergistic effect of the N-terminal and C-terminal domains of DKK1, which bind to LRP6 E1 and E3, respectively, results in a high-affinity DKK1–LRP6 interaction, with a *K*_D_ of 3 nM^2^.

SOST effectively antagonized Wnt1, Wnt2, and Wnt9b signaling, but not Wnt3a signaling (Fig. 6a). Structural alignment of LRP6 E1E2–SOST_tr177_ with LRP6 E3E4 (PDB ID 3S8Z) demonstrated that LRP6 E3E4 (which interacts with Wnt3a) could not bind SOST. Although most residues of LRP6 E1 involved in SOST binding are conserved in LRP6 E3 (Supplementary Fig. 21), structural analysis predicted that two important interactions of SOST N117 and R121 with LRP6 E3 rarely form. First, LRP6 E3 has N794 (corresponding to N185 in LRP6 E1, which interacts with N117 of SOST), but a similar interaction may be disturbed by the nearby hydrophobic residue L810, which corresponds to A201 in LRP6 E1 (Supplementary Fig. 22). Second, LRP6 E3 does not contain a residue corresponding to E73 in E1 (which forms an ionic bond with SOST R121), and instead has K684 (Supplementary Fig. 22). Regarding the SOST C-tail, since most residues constituting the ligand-binding pocket of LRP6 E2 are not conserved in LRP6 E4 (Supplementary Fig. 21), the SOST C-tail may not interact with LRP6 E4. These structural differences clearly explain previous binding-affinity measurements showing that SOST does not bind LRP6 E3E4^13^. However, Wnt3a signaling was partially inhibited by SOST (~25%) at low level of Wnt3a (Supplementary Fig. 20), which could occur if SOST binding to LRP6 E1E2 sterically interferes Wnt3a binding to LRP6 E3. Alternatively, SOST binding may induce certain conformation of LRP6 ECD that disfavors Wnt3a binding. To better understand these data, structural information for the Wnt3a–LRP6 ECD complex is needed.

Although we demonstrated that the C-terminal region of SOST is important for SOST cellular function, our in vivo data obtained using *X. laevis* embryos and cell-based assays suggest that the loop 2 region contains the primary binding site, considering that the SOST mutant with loop 2 deletion showed more severe deleterious effects on SOST function than the C-terminal truncation mutant. This model is consistent with our structural analysis of SOST bound to LRP6 E1E2. First, the SOST loop 2–LRP6 E1 interaction is more extensive than the SOST C-terminal tail–LRP6 E2 interaction (~540 Å^2^ versus ~400 Å^2^, respectively). Indeed, the average B-factor of the NXI motif of loop 2 is lower than that of the C-tail in two independent complex molecules in an asymmetric unit (Supplementary Fig. 23). Second, unlike LRP6 E1, the ligand-binding site on LRP6 E2 is not found to be freely available, unless rearrangement of the bulging-out loop occurs. Thus, LRP6 binding would occur more readily with SOST loop 2 than the C-tail. Nevertheless, our structural and functional data suggest that the E2 domain, together with the E1 and E3 domains, may serve as an important regulatory role in the Wnt signaling pathway by engaging in ligand binding.

The precise mechanism whereby SOST inhibits Wnt signaling through LRP5/6 is of great interest, as it represents a potential therapeutic target for bone diseases such as osteoporosis^18,22^. A need for a drug with high specificity and minimal side effects exists. Previously, a humanized monoclonal antibody targeting SOST was developed for treating osteoporosis in postmenopausal women^23,24^, but critical cardiovascular side effects (such as cardiac ischemia) were reported^25^. An antibody targeting both the loop 2 and C-terminal peptides may be another option for new osteoporosis drugs with better therapeutic effects and fewer side effects.

Collectively, our structural, biochemical, and in vivo findings provide an improved understanding of the regulatory mechanism of Wnt signaling in bone homeostasis and offer an important basis for the development of osteoporosis therapeutics.

## Methods

### Protein expression and purification

Genes encoding human LRP6 E1E2 (P21–P630) with a C-terminal 10× His tag and human SOST_tr177_ (Q24-S177) with a cleavable N-terminal His_6_-MBP tag were cloned into a pAcUW51 dual vector (BD Biosciences) (Supplementary Table 3) for co-expression in High Five^™^ insect cells (BTI-TN-5B1-4). Seventy-two hours after viral infection, the supernatant (containing LRP6 and SOST) was collected, filtered, and loaded onto an Ni-NTA agarose resin (Qiagen). After column washing, bound proteins were eluted with elution buffer (50 mM Tris, pH 8.0, 300 mM NaCl, 300 mM imidazole). To remove the His_6_-MBP tag, HRV3C protease was added to the eluted sample, and the mixture was incubated overnight at 4 °C. Following cleavage, the reaction mixture was loaded onto a HiTrapQ column (GE Healthcare), and the LRP6 E1E2–SOST_tr177_ complex was eluted with a linear NaCl gradient. The eluates were further purified with a size exclusion chromatography (SEC) Superdex 200 column (GE Healthcare) that was pre-equilibrated with 25 mM Tris (pH 8.5) and 200 mM NaCl. Fractions containing the LRP6 E1E2–SOST_tr177_ complex were pooled and concentrated to 3.6 mg/ml for crystallization.

For affinity measurements, all constructs (including human LRP6 E1E2, LRP6 E1 with a C-terminal 10× His tag, and human WT SOST and SOST mutants with an N-terminal His_6_-SUMO tag or N-terminal His_6_-MBP tag) were cloned into the pAcGP67A vector (BD Biosciences). Information regarding the SOST constructs is provided in Supplementary Table 1. Each construct was expressed in High Five^™^ cells and purified similarly as described above, except that no cleavage reaction with HRV 3C protease was performed. All purified proteins were concentrated to ~1 mg/ml and diluted to the appropriate concentration before use.

### Crystallization, data collection, and structure determination

For structural analysis of the LRP6 E1E2–SOST complex, we generated several SOST constructs, encoding full-length SOST, N-terminal-truncation mutants (30–213, 40–213, and 60–213), and C-terminal-truncation mutants (24–177, 24–187, 24–197, 24–203). Among these, only SOST_tr177_ (24–277) was successfully purified and crystallized in complex with LRP6 E1E2. An initial crystallization hit for the LRP6 E1E2–SOST_tr177_ complex was found in 0.1 M MOPS pH 7.5, 0.1 M magnesium acetate, and 12% PEG 8000, using a 96-well, sitting-drop plate at 298 K. Larger, but multiple long crystals were obtained in a 24-well hanging-drop plate by equilibrating 2 μl drops (consisting of 1 μl protein complex solution and 1 μl reservoir solution) against 500 μl reservoir solution, consisting of 0.1 M HEPES pH 7.2, 0.15 M magnesium acetate, 80 mM sodium citrate, and 10% PEG 8000. Initially, 4.1–4.5 Å resolution diffraction datasets were collected on beamline 11C at the Pohang Accelerator Laboratory (PAL, South Korea) and on beamline 23-ID at the Advanced Photon Source (APS, USA). After screening >200 crystals, we ultimately collected a 3.8 Å resolution dataset from beamline 23-ID of the APS, using a 10-μm diameter beam. The diffraction images were integrated and scaled using XDS suite^26^. The data collection statistics are summarized in Supplementary Table 4.

The LRP6 E1E2–SOST_tr177_ structure was solved by the MR method, using Phaser^27^. The LRP6 E1E2 structure (PDB entry 3S94) and the core cystine-knot SOST structure (PDB entry 2K8P) were used as the search models, after removing the N-terminal and C-terminal tails and the loop 2 region of SOST. The MR solution contained two independent copies of a 1:1 complex, corresponding to a solvent content of 73% (Matthews coefficient 4.5). Excess electron density was observed for the loop 2 and C-terminal HNQS regions after rigid body fitting of the MR solution. As an initial procedure during the refinement, simulated annealing was used to remove model bias. Several cycles of manual model building with COOT^28^, followed by structure refinement by PHENIX^29^ using grouped B-factors per residue and NCS restraints, were performed. Although weak electron density was observed in the SOST loop regions and C-terminal tail, the continuous electron density in a 2mFo-DFc map enabled main-chain tracing in these regions. In addition, the side chains of PNAIGR in loop 2 and HNQS in the C-terminal tail showed relatively well-defined electron density, which enabled side-chain assignments in these regions. Finally, the structure was refined with individual ADPs, which reduced the *R* factors substantially from 0.23/0.27 to 0.21/0.26. Our final model consists of residues 19–38 and 41–630 of LRP6 (chain A), 20–38 and 41–631 of LRP6 (chain B), 78–121 and 128–177 of SOST (chain C), and 78–121 and 130–177 of SOST (chain D). The final *R*_work_ and *R*_free_ values were 21% and 26%, respectively, and the Ramachandran outlier value is 0.07%. The refinement statistics are summarized in Supplementary Table 4.

### Crosslinking MS experiments

The crosslinking reaction of the purified LRP6 E1E2 H404K–SOST_tr177_ N175K complex with bis(sulfosuccinimidyl) suberate (BS3) (Thermo Scientific, Pierce, USA) was performed at room temperature for 30 min, and then quenched by 40 mM Tris-Cl (pH 7.5). The reaction mixture was heated in SDS sample buffer and loaded onto an SDS-PAGE gel. The shifted band corresponding to the molecular weight of ~90 kDa (LRP6 E1E2–SOST complex) was carefully excised for in-gel digestion by MS-grade chymotrypsin. The excised gel band was first destained and dehydrated in 50% ACN solution of 25 mM ammonium bicarbonate (ABC) buffer and then followed by in-gel reduction of disulfide bonds with 10 mM dithiothreitol in 25 mM ABC for 30 min at 56 °C. Subsequently the reduced cysteine residues were alkylated with 40 mM iodoacetamide in 25 mM ABC for 1 h at 37 °C in the dark. After three times washing out the excess reagents with 25 mM ABC, the resulting sample was digested by MS-grade chymotrypsin (Thermo Fisher) for overnight at 37 °C. The digested peptides were subjected to C18-SPE clean up using 10 µL of ZipTip (Millipore). The final peptides reconstituted with 25 mM ABC were analyzed on an Orbitrap Fusion Lumos mass spectrometer (Thermo Scientific) coupled with NanoAcquity UPLC system (Waters, Milford), which was operated at a flow rate of 300 nL/min over 1 h with linear gradient ranging from 95% solvent A (H_2_O with 0.1% formic acid) to 40% of solvent B (acetonitrile with 0.1% formic acid). Analytical capillary column (100 cm × 75 µm i.d.) and trap column (2 cm × 150 µm i.d) were packed in-house with 3 µm Jupiter C18 particles (Phenomenex). The long analytical column was placed in a dedicated 95 cm long column heater (Analytical Sales and Services) regulated to a temperature of 45 °C. Precursor ions were acquired (*m*/*z* 300–1800) at 60 K resolving power and the isolation of precursor for MS/MS analysis was performed with a 1.4 Th. Higher-energy collisional dissociation with 30% collision energy was used for sequencing with a target value of 5e4 ions determined by automatic gain control. Resolving power for acquired MS2 spectra was set to 7.5 k at *m*/*z* 200 with 22 ms maximum injection time.

The LC-MS/MS spectra were first subjected to peak picking with msConvert^30^, and then processed using MS-GF + database search algorithm^31^ (v.9979) at 10 ppm and ±0.5 Da mass tolerance for precursor and fragment ion, respectively, against the Swiss-Prot human proteome database (2019.05.07 released) containing the modified sequences for target proteins. The following search parameters were applied: semi-chymotryptic digestion, fixed carbamidomethylation on cysteine, and dynamic modification of a lysine residue (+156.079 Da) to discriminate the unmodified and dead-end modified peptides by the BS3 reagent. The number of missed cleavages cannot be specified in this search algorithm. The false discovery rate (FDR) was set to <1% for the non-redundant peptide-level (the resulting cutoff value for SpecEvalue was 7.845E−11). Protein-level FDR was not specified since most of the peptide-to-spectrum matches (>88%) were from the LRP6 E1E2–SOST complex. We identified crosslinked peptide from LRP6 E1E2 H404K–SOST_tr177_ N175K using Xcalibur Qual Browser (Thermo) among the potential list of targeted intermolecular chymotryptic crosslinks based on the identified chymotryptic peptides of LRP6 E1E2–SOST complex.

### SEC-MALS experiment

The purified LRP6 E1E2–SOST_tr177_ complex at a concentration of 1 mg/ml was injected into a Superdex 200 increase 5/150 GL column equilibrated with a buffer consisting of 50 mM Tris, pH 8.5 and 150 mM NaCl. The eluent was monitored with three detectors: a UV detector, a multi-angle laser light scattering (MALS) detector (Wyatt TREOS), and a differential refractometer detector. The collected data were analyzed using the protein conjugate method in ASTRA 6 software, distributed by Wyatt technology. The dn/dc for the LRP6 E1E2–SOST_tr177_ complex was taken to be 0.185.

### Fluorescence-SEC experiment

The purified LRP6 E1E2–Smo-SOST complex (250 µl) at a concentration of 1.5, 1, 0.5, or 0.1 µM was injected into a Superdex 200 Increase 10/300 GL column equilibrated with a buffer consisting of 20 mM Tris, pH 7.0 and 200 mM NaCl. The intrinsic tryptophan fluorescence was detected by an FP-4025 fluorescence detector (JASCO), with the excitation set at 290 nm and emission at 340 nm.

### Cell-based SOST and WISE activity assays

Construct information of the SOST and WISE mutants used for TopFlash assays are described in Supplementary Table 1. The well-established TopFlash reporter plasmid system was used to monitor activation of the canonical Wnt signaling pathway. *A Renilla* luciferase plasmid was co-transfected as a control to normalize the TopFlash luminescence signals. HEK293A cells were seeded at a density of 20,000 cells/well in a 96-well plate before transfection. The total amount of plasmid DNA was held constant as 100 ng/well (for the Wnt1- and Wnt3a-signaling assays) or 200 ng/well (for the Wnt2- and Wnt9b-signaling assays). In the latter cases, a plasmid encoding Frizzled 5 was also co-transfected. METAFECTENE® PRO (Biontex Laboratories) was used as the transfection reagent in all cases. Four hours after transfection, the medium was changed, and the cells were further incubated for 12 h. Luciferase signals were detected using the Dual-Luciferase Reporter Assay System (Promega) with an LB 96V microplate luminometer (Berthold). Three independent experiments were performed for each sample.

### Western blot and ELISA analyses

The expression levels of SOST mutants and WT SOST were compared by ELISA and western blot analyses. Each SOST construct was transfected into HEK293A cells using METAFECTENE® PRO (Biontex Laboratories). At 16 h post-transfection, the supernatants from each well (containing secreted SOST) were recovered and centrifuged to remove cell debris. Each SOST mutant and WT SOST in the supernatants were quantified using a Quantikine ELISA Kit (R&D Systems, Catalog #DSST00). The expression levels of WISE mutants were compared to that of WISE WT using an ELISA Kit (MyBioSource, Catalog #MBS9308697).

Since the SOSTΔloop2 mutant was not detected by the corresponding primary antibody of the Quantikine ELISA Kit (R&D Systems), its expression was confirmed by western blotting. Supernatants containing SOSTΔloop2 and WT SOST were prepared as described above. Each sample was concentrated to the same volume, loaded into an SDS-PAGE gel, and visualized by western blot using a monoclonal SOST antibody (Invitrogen, Catalog #MA5-23897, 1:1000 dilution).

Anti-GAPDH antibody (Santa Cruz Biotechnology, Catalog #sc-47724, 1:1000 dilution) was used as a loading control for western blots and anti-His-tag antibody (Cell Signaling, Catalog #2366, 1:1000 dilution) was used for western blots in fluorescence-SEC experiments (Supplementary Fig. 11).

### Microscale thermophoresis

Affinity measurements were performed using a Monolith NT.115 Pico instrument (NanoTemper). Purified LRP6 E1E2 and LRP6 E1 were labeled with the dye NT-647 using the Monolith NT Protein Labeling kit (NanoTemper). Labeled LRP6 E1E2 and E1 were used at concentrations of ~ 5 nM. Each unlabeled Smo-SOST (WT and mutants), MBP-SOST (WT), and peptides were diluted with MST buffer (20 mM Tris-Cl, pH 7.0, 200 mM NaCl, 0.5 mg/ml BSA, and 0.05 (v/v) % Tween 20). After 10 min incubation of each mixture at room temperature, each sample was filled into Monolith NT.115 standard capillary. The measurements were performed at 5% LED power and 40% MST power at 22 °C. MST data were analyzed by non-linear regression with the specific binding of Prism software.

### Preparation of SOST peptides and histone peptide for affinity measurements

DNA encoding the human SOST C-terminal tail (residues 170–213) and a region of human histone H3 (residues 1–44) were subcloned into the pGEX-4T-1 vector (GE Healthcare). Each peptide was expressed in *E. coli* strain Rosetta (DE3) with an HRV3C-cleavable GST tag. After overnight induction with 0.2 mM isopropyl β-d-1 thiogalactopyranoside at 20 °C, the cells were harvested and lysed with an Emulsiflex C3 device (Avestin). Each lysate was clarified via centrifugation, and the supernatant was loaded onto a glutathione agarose column (Thermo Scientific). After column washing with equilibration buffer (20 mM HEPES, pH 7.0 and 150 mM NaCl), each GST-tagged peptide was eluted with 20 mM reduced glutathione in equilibration buffer. After cleaving the GST tags with HRV 3C protease, ion-exchange chromatography with a HiTrapSP column (GE Healthcare) and size-exclusion chromatography with a Superdex 200 10/300 GL column (GE Healthcare) were performed. The purified SOST C- tail and histone peptides were concentrated and used for affinity measurements.

A disulfide-bonded, circularized loop 2 peptide (CLLPNAIGRGKWGC) was synthesized (Peptron), dissolved in MST buffer, and used for affinity measurements.

### Modeling of the SOST C-terminal tail

Candidate structures of the SOST C-terminus (residues 178–204) bound to LRP6 E2 were generated based on the crystal structure of LRP6 E1E2–SOST_tr177_ by initial modeling and docking of the following two underlined peptide sequences in the 178–204 region of SOST (178-ELKDFGTEAARPQKGRKPRPRARSAKA-204), which was followed by modeling the remaining residues and the overall structure relaxation.

The resolved C-terminal structure of SOST, ending at residue 177, was extended via terminus modeling residues 178–183 with GalaxyLoop software^32^, after observing exposed hydrophobic residues on the surface of LRP6 near SOST 177. Indeed, SOST L179 and F182 fit well into the hydrophobic core with F536, W550, and M576 of LRP6. Next, the well-conserved, positively charged fragments (188-RPQKGRKPR-196 or 188-RPQKGRK-194) were docked on the surface of LRP6 using PIPER-FlexPepDock software^33^ and GalaxyPepDock-ab-initio^34^, respectively. The shorter peptide was used for GalaxyPepDock-ab-initio docking because, in our experience, shorter peptide generally show better performance. The PIPER-FlexPepDock program was run separately on LRP6 E1 and LRP6 E2 (split at LRP6 T312) because of the protein-size limit. GalaxyPepDock-ab-initio analysis was performed for the LRP6 E1E2 structure. The docked structures generated using both methods were evaluated by simulating the connection of G183 to the linker 184-TEAA-187 of SOST. To identify plausible candidate structures, the linker 184-TEAA-187 and the remaining terminal residues were modeled by GalaxyLoop. Redundant model structures were removed after structure clustering, based on root-mean-square-distances. Lastly, non-redundant structures were relaxed using GalaxyRefineComplex^35^, and two final structures with the best GALAXY energy^35^ were selected (representatively model 1, illustrated in the right panel of Supplementary Fig. 12). These structures showed multiple hydrogen bonds and electrostatic interactions between the C-terminal residues of SOST and those of LRP6 E2.

### Clustered regularly interspaced short palindromic repeats (CRISPR)-based LRP6 targeting

LRP6-null HEK293T cells were generated using the CRISPR/CRISPR-associated protein 9 genome-editing method^36^. Briefly, a guide RNA targeting the sequence 5′-TTGACAGCCACAAATCCATGTGG-3′ in exon 5 of human LRP6 was cloned into the lentiCRISPRV2 vector (Addgene plasmid #49535) by annealing the following oligos and ligating them into the *Bsm*BI sites: Fwd, 5′-CACCGTTGACAGCCACAAATCCATG-3′; Rev, 5′-AAACCATGGATTTGTGGCTGTCAAC-3′. Lentiviral gene delivery was performed as described^37^. Infected cells were selected using puromycin (2 μg/ml; Clontech) for 2 days. Eight clones were isolated from a single cell, expanded, and analyzed by western blotting with a monoclonal human LRP6 antibody (Cell Signaling, Catalog #3395S, 1:1000 dilution). One clone was confirmed to be an LRP6 knockout cell line and was used for TopFlash assays with the LRP6 mutant.

### *Xenopus laevis* embryos and microinjection

*X. laevis* embryos were purchased from Nasco (USA) and used following the instructions from the POSTECH Institutional Animal Care and Use Committees (Korea), after obtaining certification for ethical handling. Eggs were obtained from female frogs primed with 800 U of human chorionic gonadotropin (Sigma). In vitro fertilization was performed in 33% Modified Ringer (MR) solution. The developmental stages of the embryos were determined according to Nieuwkoop and Faber^38^. Microinjection was conducted in 33% MR containing 4% Ficoll-400 (GE Healthcare) using a PLI-100 Pico-Injector (Harvard Apparatus). For all capped mRNAs, the constructs were digested with *Not*I, and then the mRNAs were synthesized in vitro using the mMessage mMachine Kit (Ambion). Injected embryos were cultured in 33% MR until stage 8 and then transferred to 10% MR, until they reached the appropriate stage for the experiments. For each axis-duplication assay, the in vitro-synthesized mRNAs were introduced into a single ventro-vegetal blastomere at stage 3. Together with 30 pg *Wnt1* mRNA, 25 pg of different *SOST* mRNAs (WT *SOST*, *SOST*_*tr169*,_ *SOST*_*tr177*,_ *SOST*_*tr204*_, and *SOST∆loop2* mRNA) were injected. The duplicated axes were monitored at stage 19. For the animal cap assays, animal cap tissues were dissected at stage 8 and then cultured to stage 11. *P* values were determined by performing a two-tailed *t*-test.

### Real-time qPCR analysis

Total RNA was isolated from dissected animal cap tissues using the TRIZOL reagent (Invitrogen), and complementary DNA was synthesized using M-MLV Reverse Transcriptase (Promega) with an oligo(dT) 18 primer, according to the manufacturer’s protocol. Real-time qPCR was performed using the StepOne Real-Time PCR system (Applied Biosystems) and SYBR Green PCR Master Mix (Applied Biosystems), and the relative quantities were analyzed by the comparative Ct method described in the manufacturer’s manual. The following thermocycling conditions were used for PCR: 95 °C (10 min) and 40 cycles of 95 °C (30 s) and 60 °C (1 min). The sequences of the primers used were as follows: ODC forward 5′-TGCACATGTCAAGCCAGTTC-3′, ODC reverse 5′-GCCCATCACACGTTGGTC-3′; siamois forward 5′-TCTGGTAGAACTTTACTCTGTTTT-3′, siamois reverse 5′-AACTTCATGGTTTTGCTGACC-3′; and nodal 3.1 forward 5′-CCAAAGCTTCATCGCTAAAAG-3′, nodal 3.1 reverse 5′-AAAAGAAGGGAGGCAAATACG-3′.

### Reporting summary

Further information on research design is available in the Nature Research Reporting Summary linked to this article.

## Supplementary information

Supplementary Information

Reporting Summary

## Data Availability

The coordinate and structure factor have been deposited in the PDB under accession number of 6L6R. Other data supporting the findings of this work are available from the corresponding author upon reasonable request. Source data are provided with this paper.

## Source data

Source Data
