# Author Correction: Sclerostin inhibits Wnt signaling through tandem interaction with two LRP6 ectodomains

**DOI:** 10.1038/s41467-022-28394-6

**Published:** 2022-02-02

**Authors:** Jinuk Kim, Wonhee Han, Taeyong Park, Eun Jin Kim, Injin Bang, Hyun Sik Lee, Yejin Jeong, Kyeonghwan Roh, Jeesoo Kim, Jong-Seo Kim, Chanhee Kang, Chaok Seok, Jin-Kwan Han, Hee-Jung Choi

**Affiliations:** 1 Department of Biological Sciences, Seoul National University, Seoul, 08826 Republic of Korea; 2 Department of Life Sciences, Pohang University of Science and Technology, Pohang, Gyeongbuk 37673 Republic of Korea; 3 Department of Chemistry, Seoul National University, Seoul, 08826 Republic of Korea; 4 School of Pharmacy, Sungkyunkwan University, Suwon, 16419 Republic of Korea; 5 Center for RNA Research, Institute for Basic Science, Seoul, 08826 Republic of Korea; 6 Present Address: Plumbline Life Sciences, Inc., Seoul, 06552 Republic of Korea; 7 Present Address: Department of Physiology and Cellular Biophysics, Columbia University Irving Medical Center, New York, NY 10032 USA

**Keywords:** Intracellular signalling peptides and proteins, X-ray crystallography

Correction to: *Nature Communications* 10.1038/s41467-020-19155-4, published online 23 October 2020.

In this article the author name Yejin Jeong was incorrectly written as Yejing Jeong. The original article has been corrected.

