# Supplementary material for: Sclerostin inhibits Wnt signaling through tandem interaction with two LRP6 ectodomains
Source: Nat Commun. 2020 Oct 23;11:5357. doi: 10.1038/s41467-020-19155-4 (PMC7585440; doi:10.1038/s41467-020-19155-4)
Supplement: Supplementary file 1 — Supplementary Information [file 41467_2020_19155_MOESM1_ESM.pdf]

Supplementary Information

Sclerostin inhibits Wnt signaling through tandem interaction with two LRP6 ectodomains

Jinuk Kim, Wonhee Han, Taeyong Park, Eun Jin Kim, Injin Bang, Hyun Sik Lee, Yejing Jeong, Kyeonghwan Roh, Jeesoo Kim, Jong-Seo Kim, Chanhee Kang, Chaok Seok, Jin-Kwan Han, Hee-Jung Choi

Supplementary Figures (1 – 23)

Supplementary Tables (1 – 4)

Supplementary References

Supplementary Figure 1 Initial difference map for SOST loop2 and C-tail regions.

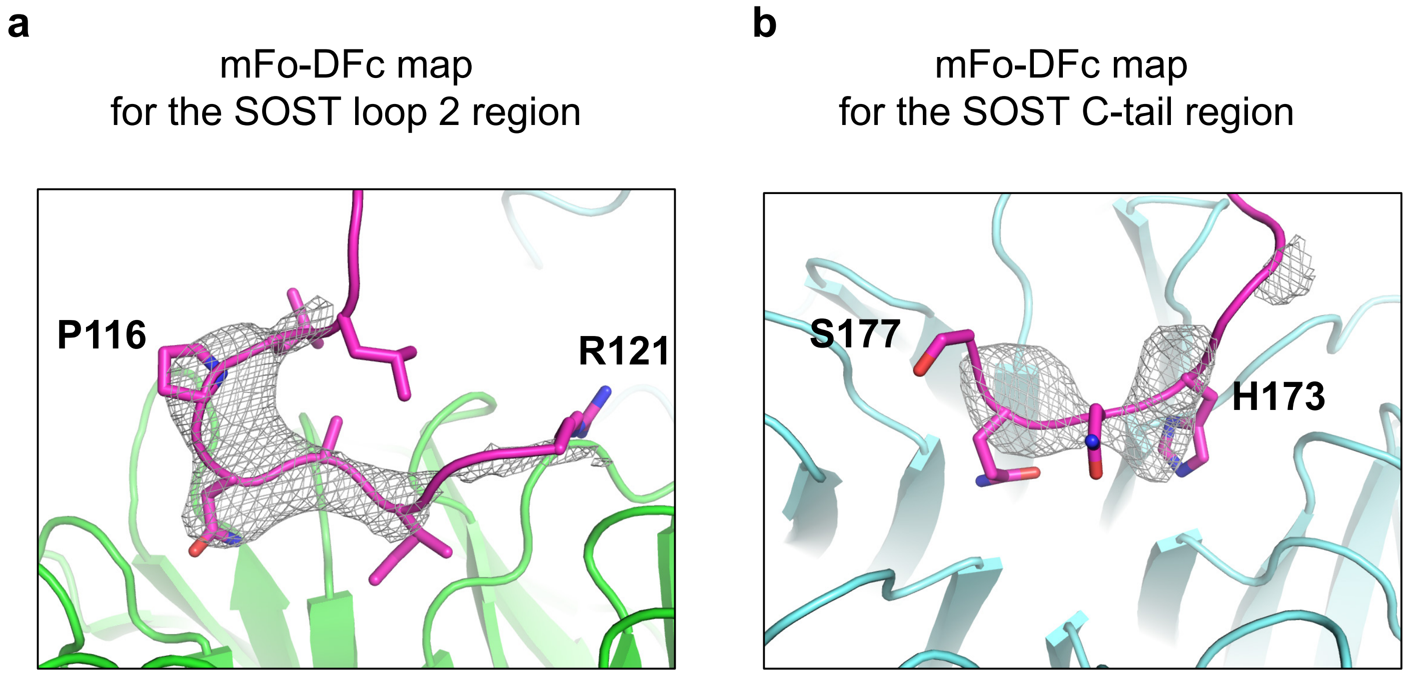

The final SOST model is shown in purple for reference. (a) The initial mFo - DFc map corresponding to the SOST loop2 and (b) SOST C-tail regions contoured at 3σ density level is shown in the grey mesh.

Supplementary Figure 2 SEC-MALS analysis of LRP6 E1E2-SOST$_{tr177}$ complex.

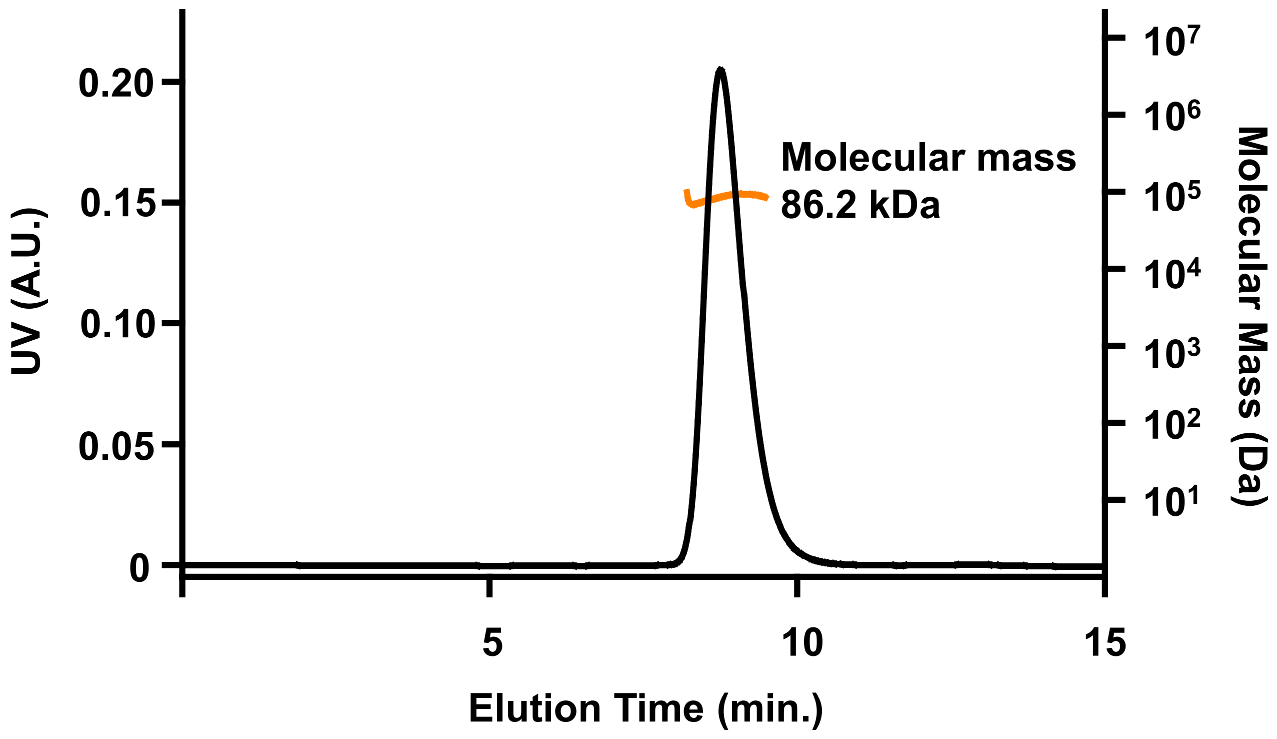

SEC-MALS profile of the LRP6 E1E2-SOST$_{tr177}$ complex is shown. The orange line represents the measured molar mass. The left and right y-axes indicate UV$_{280}$ signal and molar mass, respectively. The calculated molecular mass of the complex is 92.5 kDa.

Supplementary Figure 3 2mFo-DFc map and composite map showing the SOST loop 2 and C-tail regions.

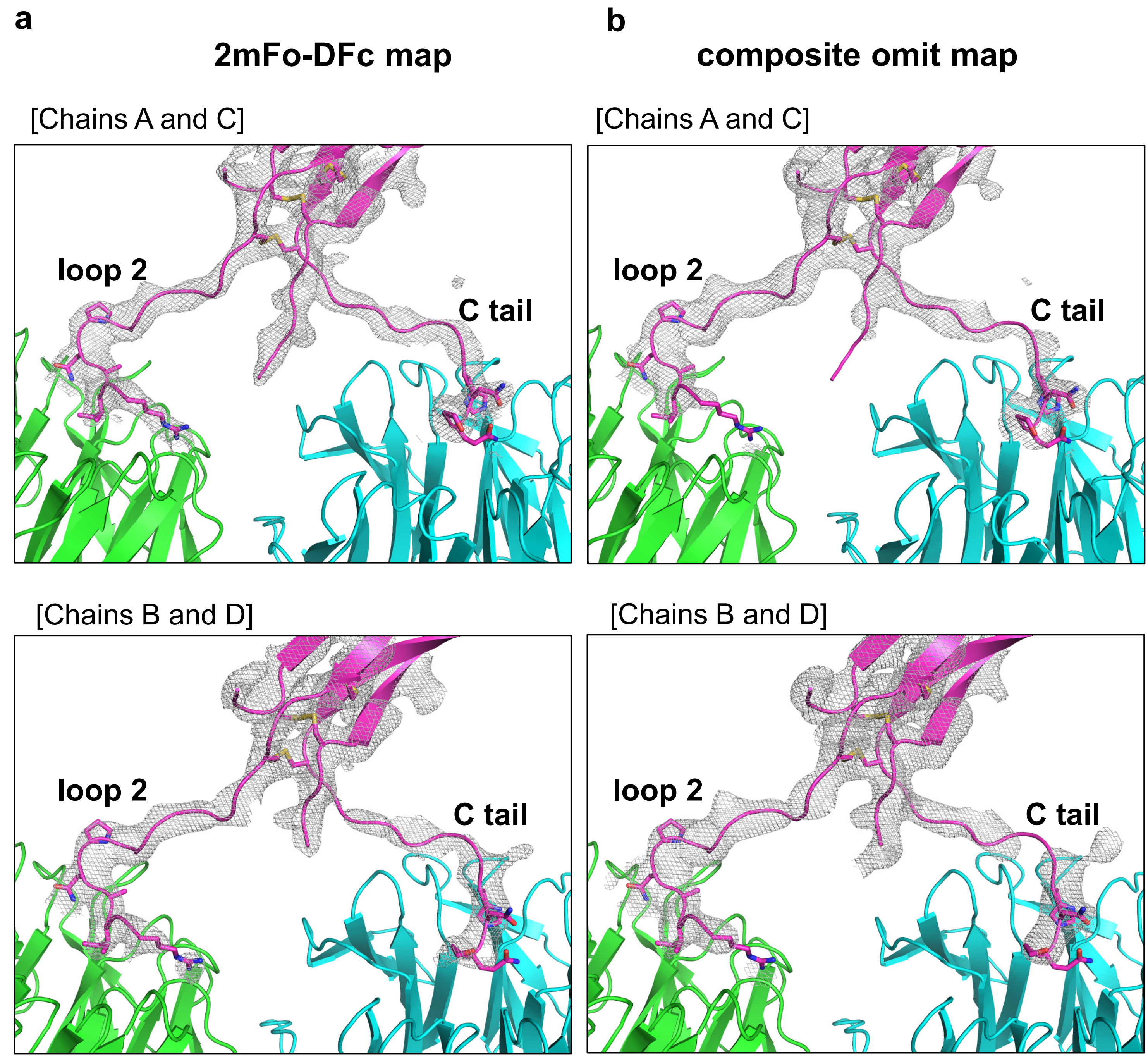

The final model of the LRP6 E1E2-SOST$_{tr177}$ complex is shown as ribbon diagram. (a) Grey meshed 2mFo-DFc map and (b) composite omit map (simple omit map calculated by the program PHENIX) contoured at 1.0 σ level are shown for the SOST loop 2 and C-tail regions. The C-terminal part of the SOST loop 2 region (122-127 in chain C, 122–130 in chain D) is disordered. Side-chains of PNAIGR in loop 2 and HNQS in the C-tail are represented as sticks. The fragmented electron density for the C-tail was observed in chain D, resulting in lower quality of fit into electron density map.

Supplementary Figure 4 Crystal packing showing the LRP6 E1E2-SOST$_{tr177}$ complex lattice.

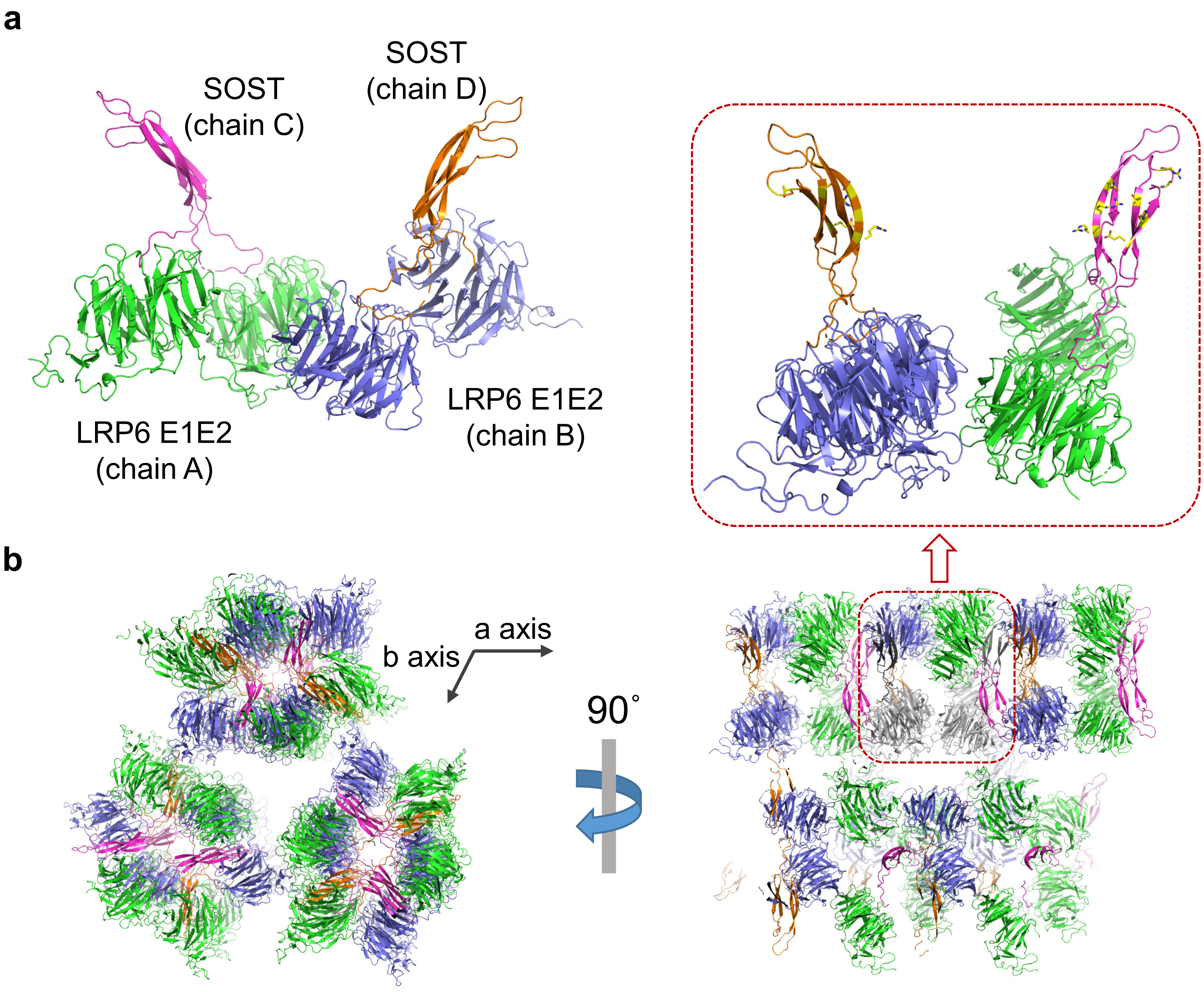

(a) There are two complex molecules in an asymmetric unit of the crystal lattice, corresponding to a solvent content of 73% (Matthews coefficient[1] 4.5). (b) Crystal packing showing the LRP6 E1E2-SOST$_{tr177}$ complex lattice is presented in two orientations. Crystallographic a and b axes are represented and c axis, which is vertical to the ab plane is omitted. On the right, the two complex molecules in an asymmetric unit are shown in grey (light grey for LRP6 E1E2 and dark grey for SOST) inside a red box. These molecules are enlarged and shown above. In a zoomed view, the SOST residues that interact with neighboring molecules within 4 Å distance, are shown in yellow stick. SOST loop 2 and C-tail are not involved in the direct crystal contact.

Supplementary Figure 5 Mass Spectrum analysis of the cross-linked peptide.

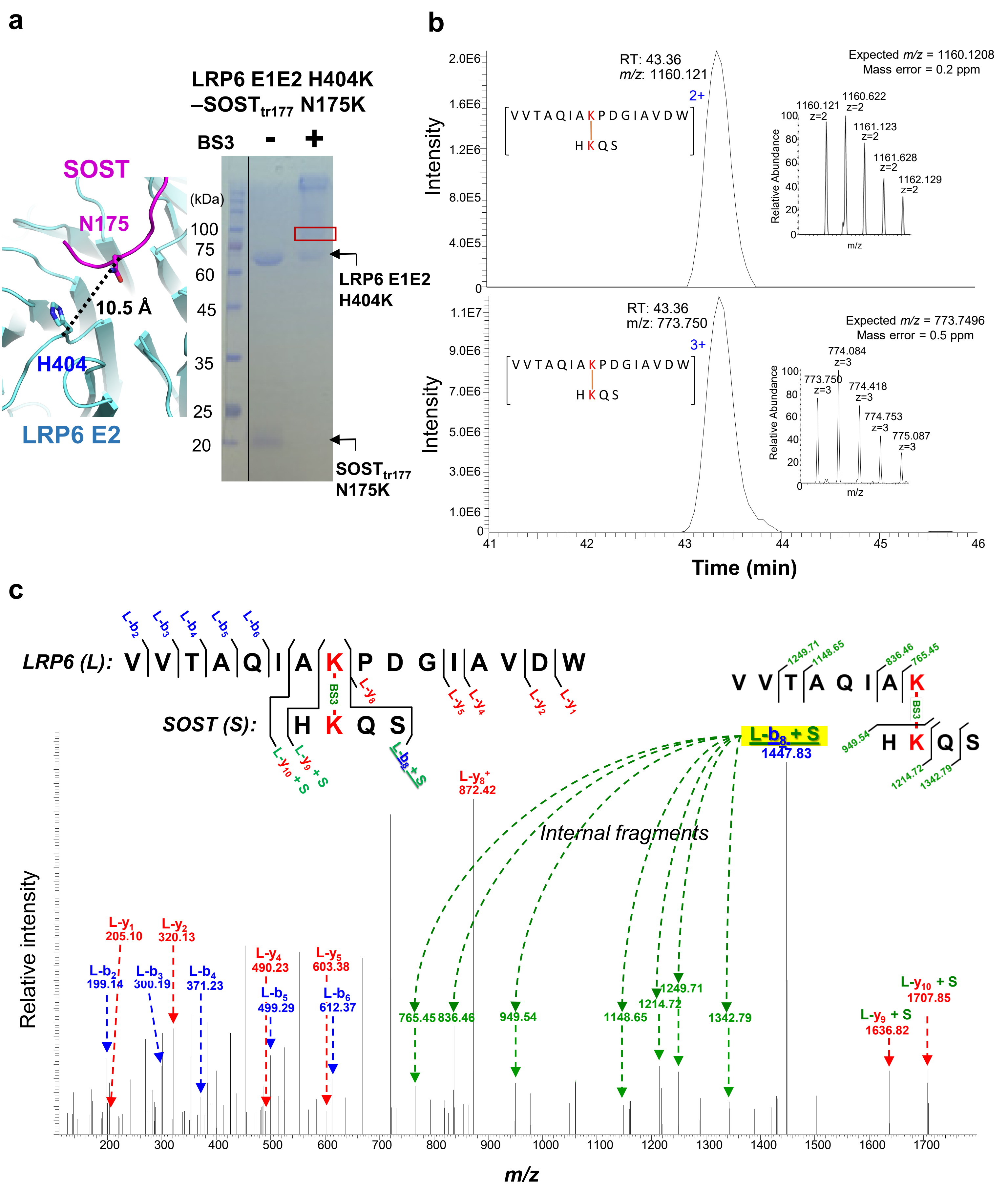

(a) Based on the crystal structure of the LRP6 E1E2 –SOST$_{tr177}$ complex, H404K and N175K mutations were introduced into LRP6 E1E2 and SOST$_{tr177}$, respectively. The shifted band corresponding to the crosslinked sample is marked with a red box. Molecular weight markers in kDa are shown on the left. Experiments were performed three times and one representative data is shown. (b) Extracted ion chromatograms of the doubly or triply charged peptide crosslink precursor ions, which are evidently co-eluted with highly accurate mass measurement as the high resolution mass spectra corresponding to the respective precursor ions are shown together. (c) Annotated tandem mass spectrum for the doubly charged crosslink ion. The internal fragment ions from the most intense 'L-B8+S' ion were specified as green color.

Supplementary Figure 6 Structural comparison of SOST in the absence and the presence of LRP6

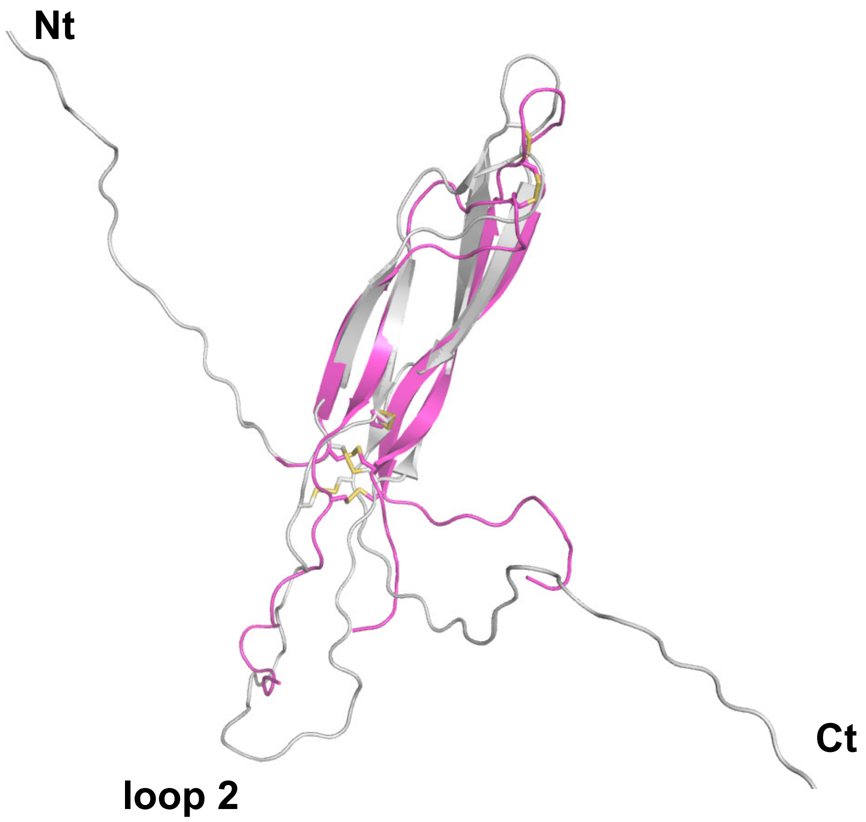

One representative SOST NMR model (PDB ID: 2K8P) and $SOST_{tr177}$ of our complex structure are compared by the structural alignment of cystine knot core. Aligned NMR structure and $SOST_{tr177}$ structure are colored light gray and magenta, respectively. Disulfide bonds are represented as yellow sticks.

Supplementary Figure 7 Deglycosylation of SOST by PNGase F.

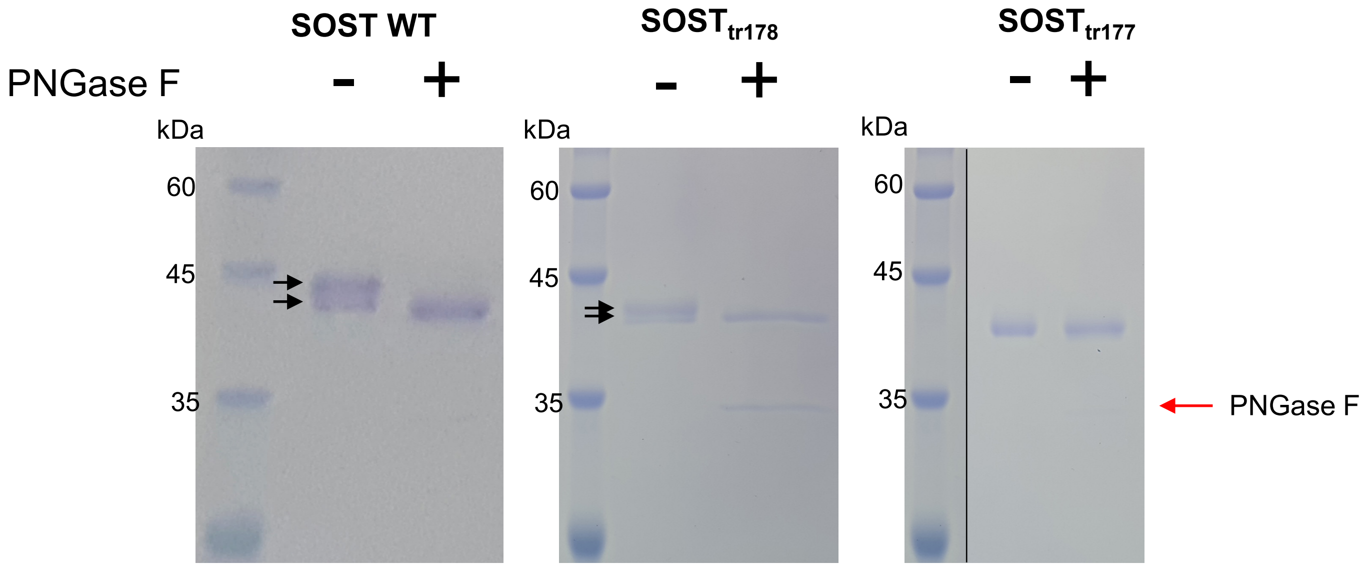

In contrast to SOST$_{tr177}$, purified SOST WT and SOST$_{tr178}$ were detected as doublets (black arrows) in an SDS-PAGE gel. To verify that each doublet was caused by glycosylation, deglycosylation reaction was carried out by PNGase F (New England Biolabs) treatment. Each reaction mixture was incubated at 37°C for 1 hour and the sample was analyzed by SDS-PAGE. Molecular weight markers in kDa are shown on the left. Experiments were performed twice and one representative data is shown.

Supplementary Figure 8 The model of N175-linked N-acetyl-β-D-glucosamine (NAG).

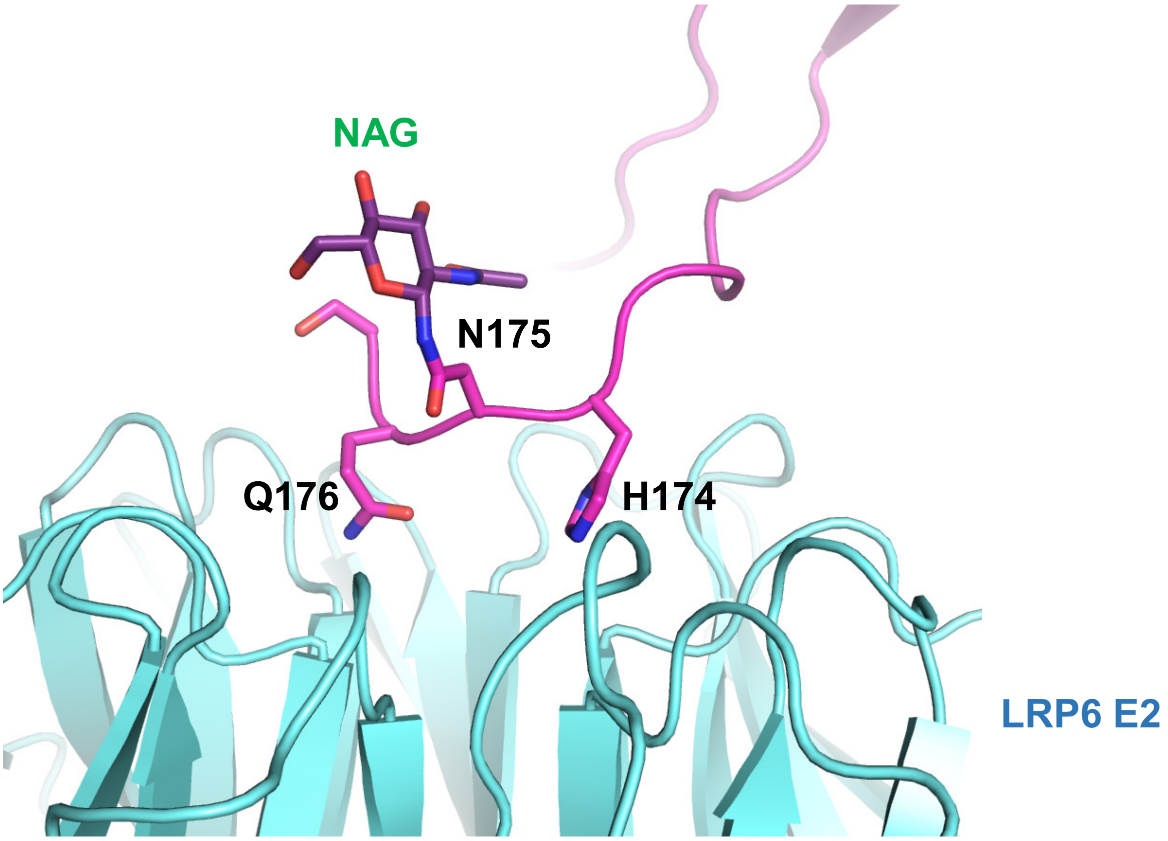

The model of N175-linked N-acetyl-β-D-glucosamine (NAG) was built by COOT[3]. Although only the first glycan (NAG-Asn) was modeled, glycosylation at N175 doesn't appear to interfere with LRP6 binding, since NAG is directed toward the solvent.

Supplementary Figure 9 Sequence alignment of SOST, WISE, and DKK1.

```
                            .         .         .         .         .
hSOST    24   QGWQAFKNDATEIIPELGEYPEPPPELENNKTMNRAENGGRPPHHPFETK   73
hWISE    24   -----FKNDATEILYSHVVKPVPAHPSSNSTLNQARNGGRHFSNTGLDRN   68

                            .         .         .         .         .
hSOST    74   DVSEYSCRELHFTRYVTDGPCRSAKPVTELVCSGQCGPARLLPNAIGRGK  123
hWISE    69   TRVQVGCRELRSTKYISDGQCTSISPLKELVCAGECLPLPVLPNWIGGGY  118
hDKK1    38                                            NSNAIKNLP   46

                                .         .         .         .
hSOST   124   ----WWRPSGPDFRCIPDRYRAQRVQLLCPGGEAPRARKVRLVAS-CKCK  168
hWISE   119   GTKYWSRRSSQEWRCVNDKTRTQRIQLQCQDGSTR-TYKITVVTA-CKCK  166

                       .         .             .         .         .
hSOST   169   RLTRFHNQSELKDFGT-EAARPQ---KGRKPRPRARSAKANQAELENAY   213
hWISE   167   RYTRQHNESS-HNFESMSPAKPVQHHRERK-RAS-KSSKHSMS         206
```

Sequence alignment of human SOST, WISE and DKK1 was performed by the CLUSTALW program[2]. The red-colored Cys residues form disulfide bonds and the conserved PNXIG residues are colored green. HNQ(E)S motif in SOST and WISE are presented in bold and the basic residues at C-tail were colored in blue. In the case of DKK1, only the N-terminal NXI motif region is shown.

Supplementary Figure 10 Affinity measurement of LRP6 E1E2 and E1 with SOST C-tail truncation mutants by MST.

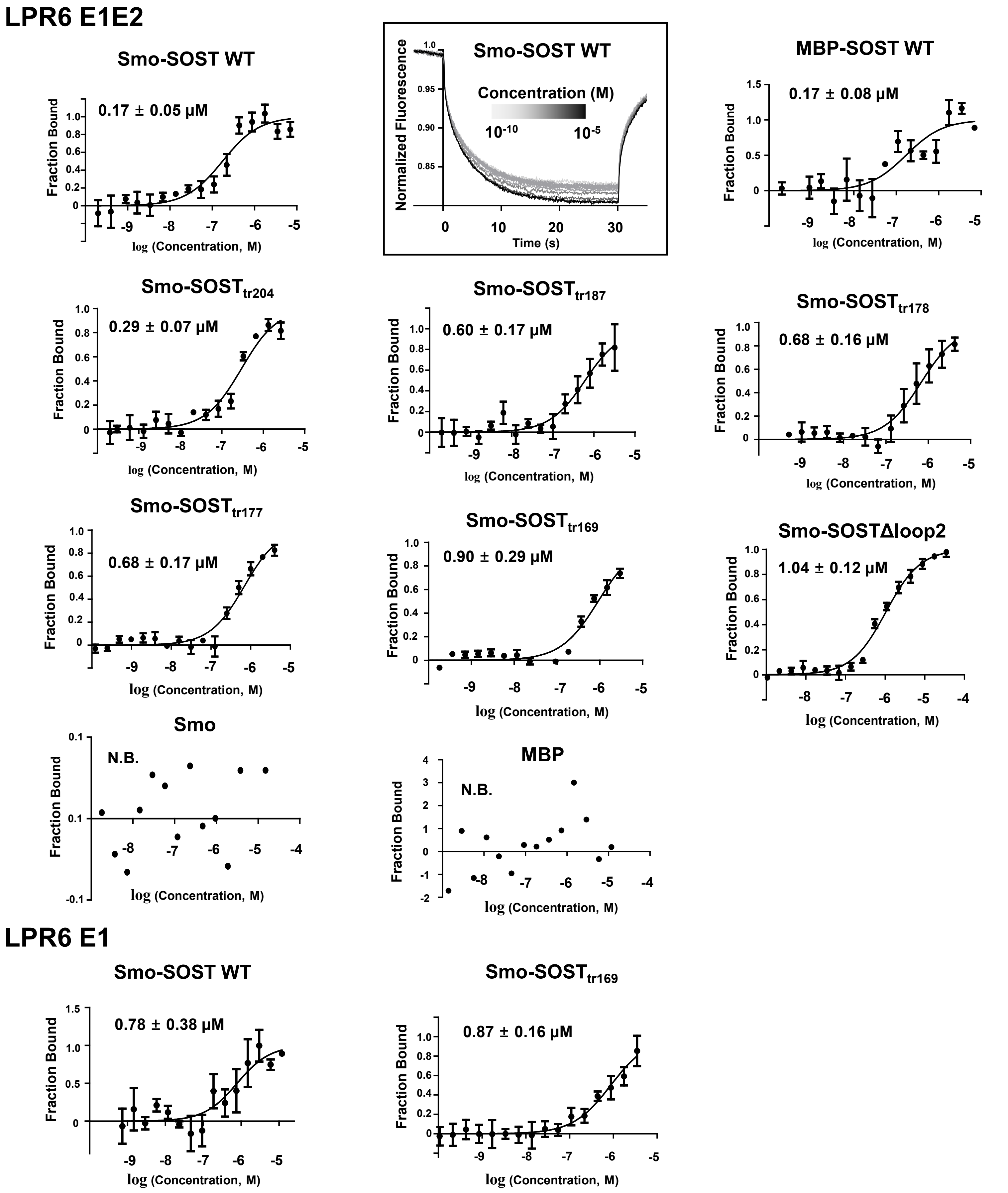

Cy-5 dye-labeled LRP6 E1E2 or LRP6 E1 was titrated with each SOST protein. Each binding curve was obtained by Graphpad Prism. Data from three independent experiments (n=3) were analyzed and expressed as mean ± SD. The calculated K_D value for each binding is shown and summarized in supplementary Table 2. The SUMO tag (Smo) and MBP tag (MBP) showed no binding to LRP6 E1E2 (N.B.; No apparent binding). As a representative, the raw MST trace of Smo-SOST WT binding to LRP6 E1E2 over time is shown in the box. The titrated concentrations are represented as the gradient lines.

Supplementary Figure 11 The FSEC profiles of LRP6 E1E2 in complex with SUMO-tagged SOST (Smo-SOST) at various concentrations.

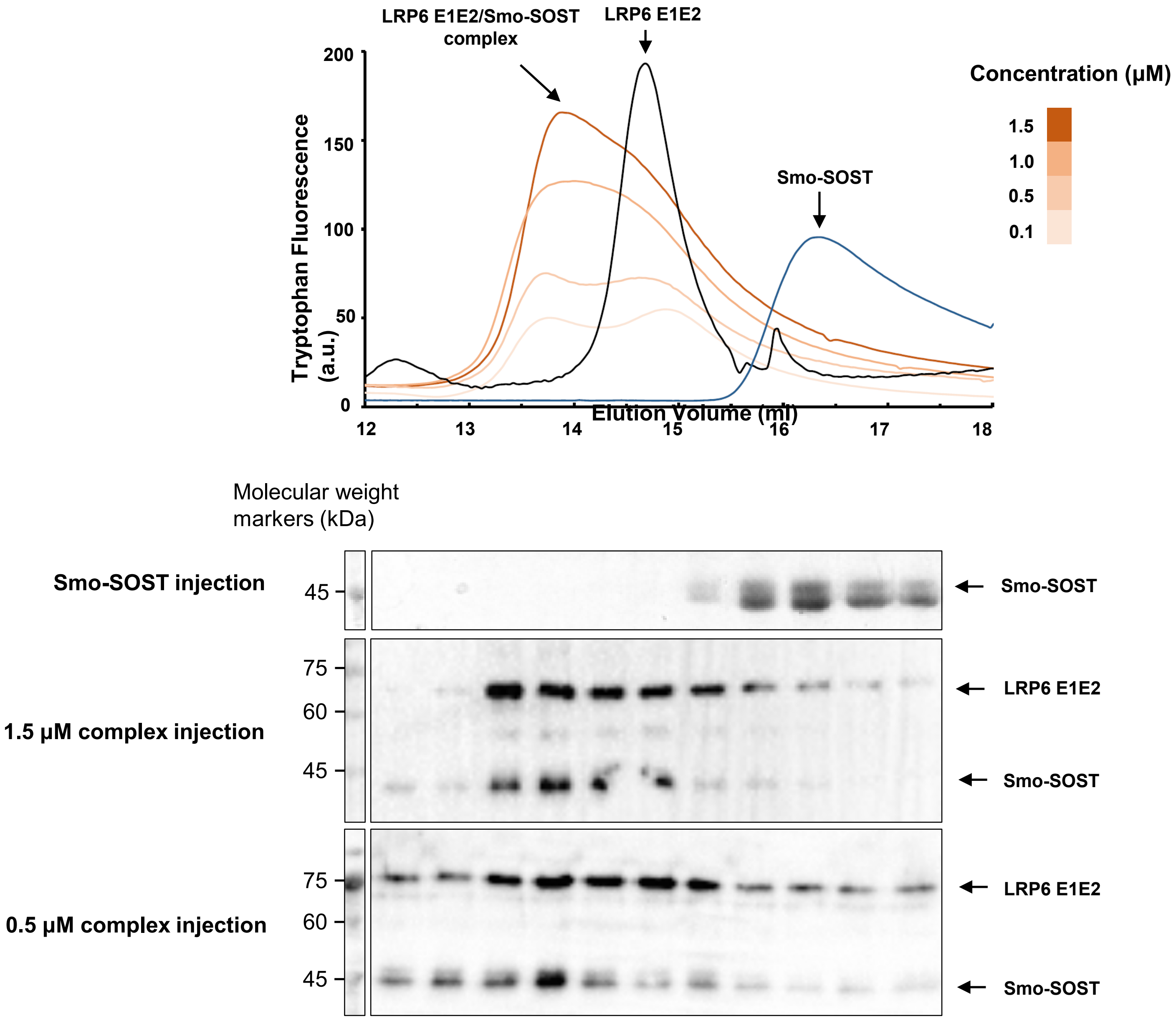

Purified LRP6 E1E2 – Smo-SOST complex was diluted to 1.5 µM, 1.0 µM, 0.5 µM, and 0.1 µM and each sample was loaded onto the SEC column. SEC profiles at each concentration were overlaid, together with those of LRP6 E1E2 (dark grey) and Smo-SOST (blue) as references. The lighter the FSEC profile of the complex, the lower the concentration as shown in the color code on the right. When the complex was diluted to 0.5 µM and injected to the SEC column, the complex appears to be half-dissociated, suggesting that the estimated dissociation constant (K_D) would be ~ 50 nM, considering the dilution factor of 10 during the SEC column. Eleven elution fractions (elution volume from 12 to 17 ml) from each SEC at 1.5 µM and 0.5 µM concentrations were visualized by western blot using HRP conjugated His6 antibody (Cell Signaling). Western blots are representative of three independent experiments.

Supplementary Figure 12 Modeling of SOST C-tail in complex with LRP6 E1E2.

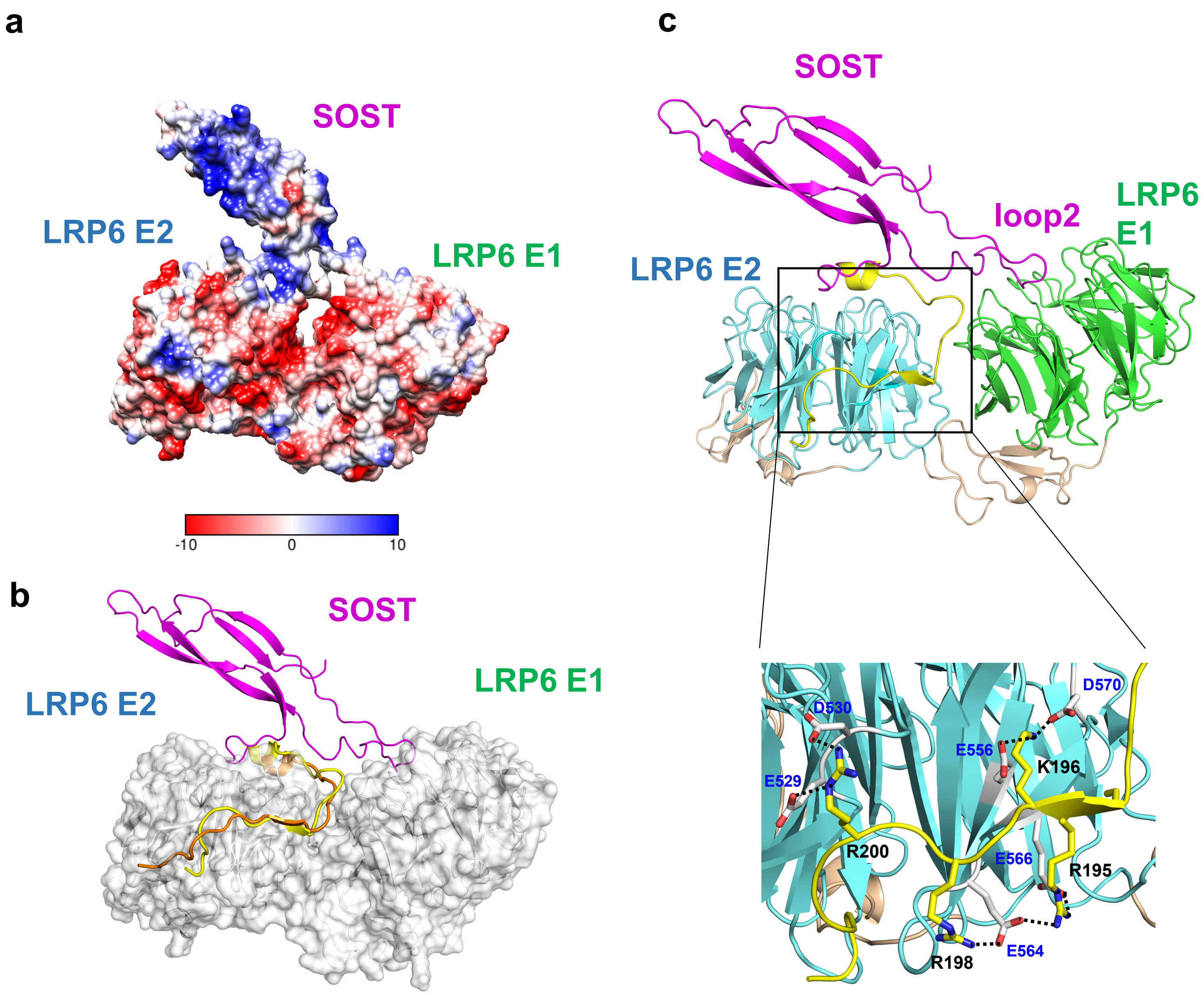

(a) The surface electrostatic potential of the crystal structure of the LRP6 E1E2-SOST$_{tr177}$ complex was calculated by the APBS program[3] and displayed using Chimera[4]. Blue and red represent the positively and negatively charged regions, respectively. (b) Two representative structures of SOST C-tail bound to LRP6 E2 with the best energy from modeling are shown as yellow and orange, respectively. LRP6 E1E2 are represented as surface model using PyMol program[5]. (c) As a representative, model 1 structure of SOST in complex with LRP6 E1E2 from modeling is shown. Residues involved in the interaction between C-tail and LRP6 E2 are represented as sticks in a zoomed view on the bottom. Interacting residues are labeled in black (SOST C-tail) and blue (LRP6 E2), respectively.

Supplementary Figure 13 MST data for the interaction between LRP6 and SOST peptides.

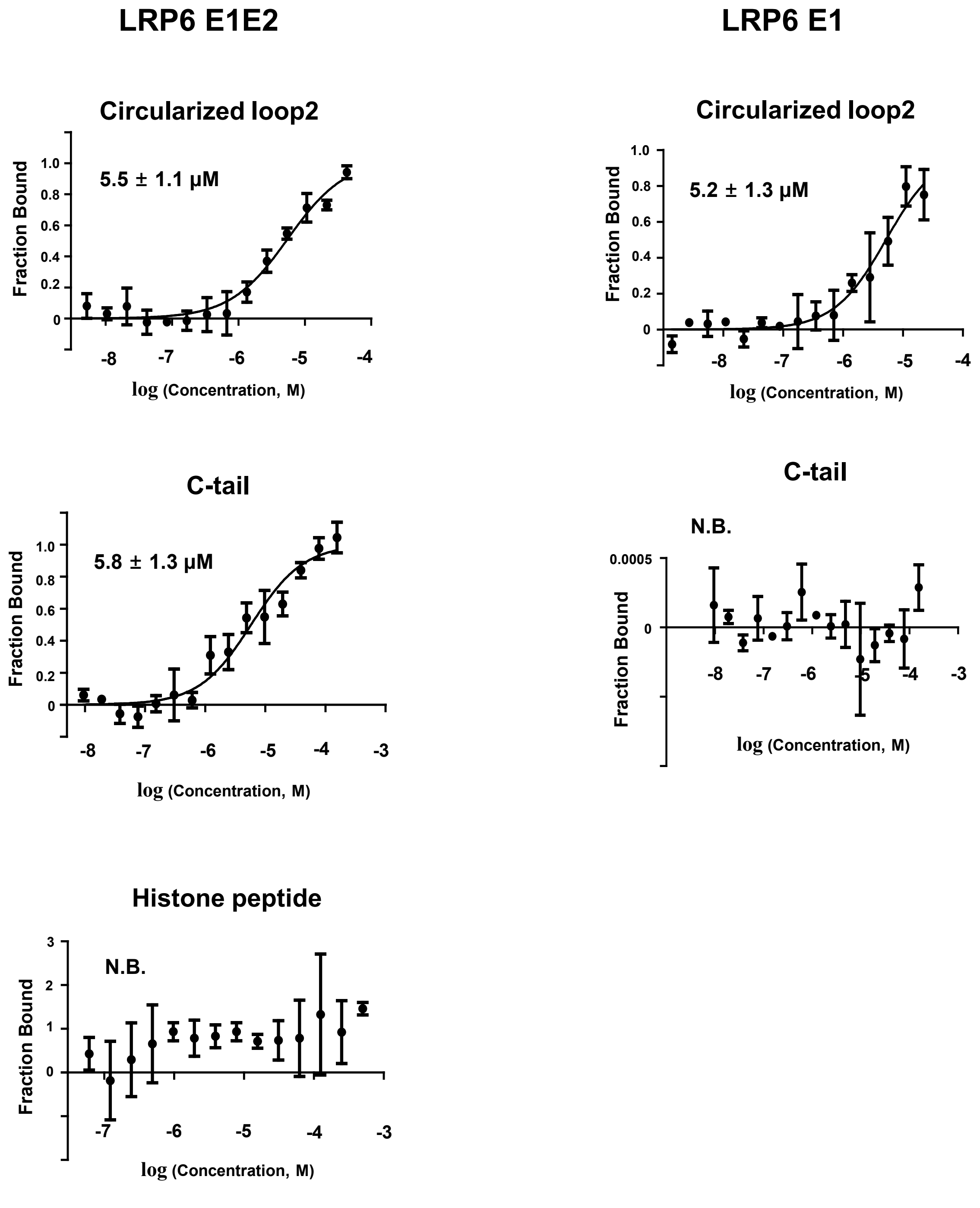

Cy-5 dye-labeled LRP6 E1E2 or LRP6 E1 was titrated with each SOST peptide. Sequence information about circulized loop 2 and C-tail peptides is described in the Online Method and Supplementary Table 1. Histone peptide (1-44) was used as a negative control. Each binding curve was obtained by Graphpad Prism and the calculated K_D values are presented. Data from three independent experiments (n=3) were analyzed and expressed as mean ± SD. No apparent binding (N.B.) was detected in the reactions of histone peptide with LRP6 E1E2 and C-tail peptide with LRP6 E1.

Supplementary Figure 14 Expression of SOST and WISE (WTs and mutants) detected by ELISA and western blot.

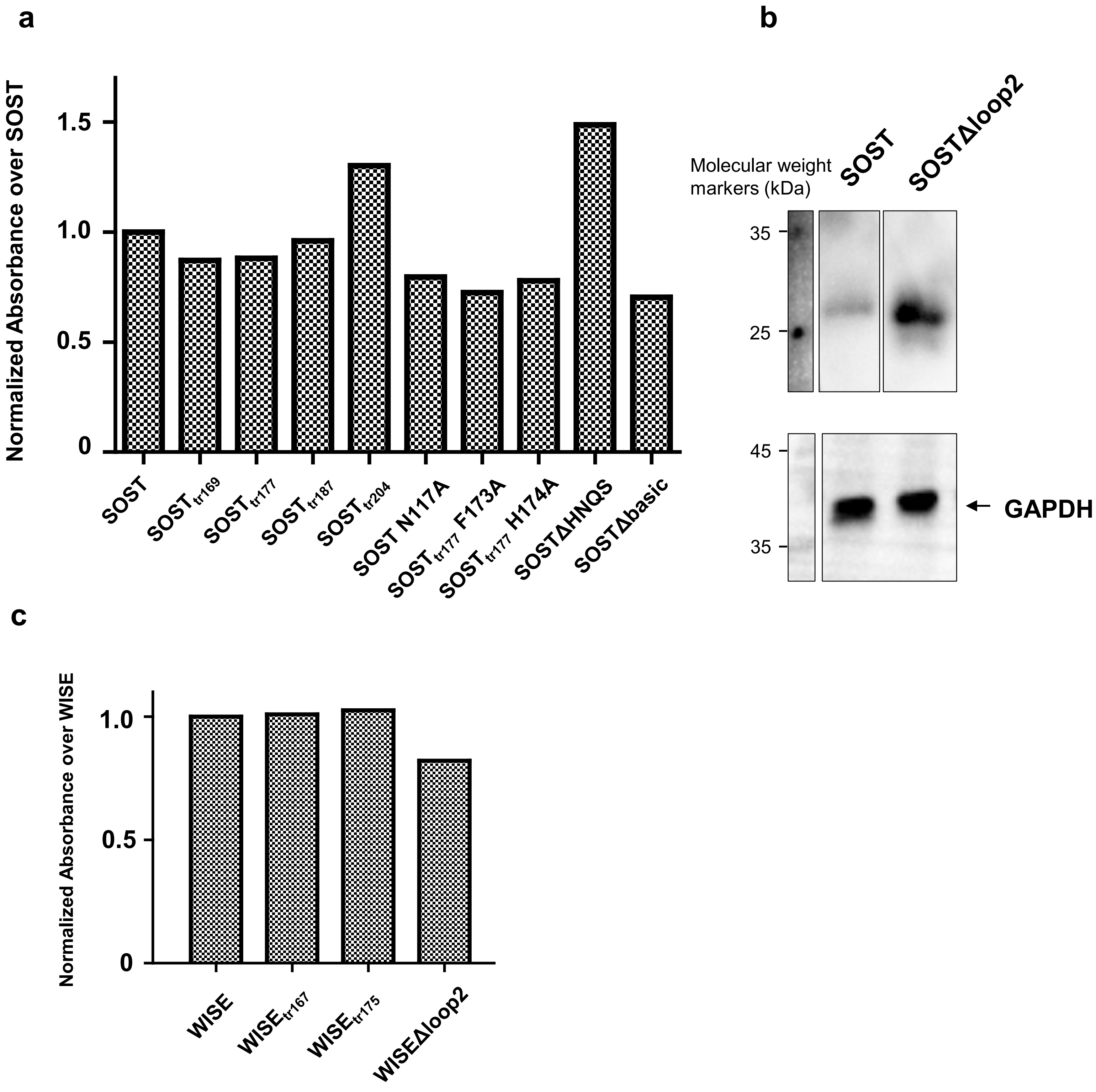

(a) The relative expression level of each SOST mutant compared to WT SOST was performed by ELISA or (b) western blot using the monoclonal SOST antibody (Invitrogen). Western blots are representative of two independent experiments. (c) The relative expression level of each WISE mutant compared to WT WISE was performed by ELISA. ELISA experiments were performed three times and one representative data are shown in (a) and (c).

Supplementary Figure 15 Effect of H174A mutation of $SOST_{tr177}$ on Wnt1-signaling inhibition.

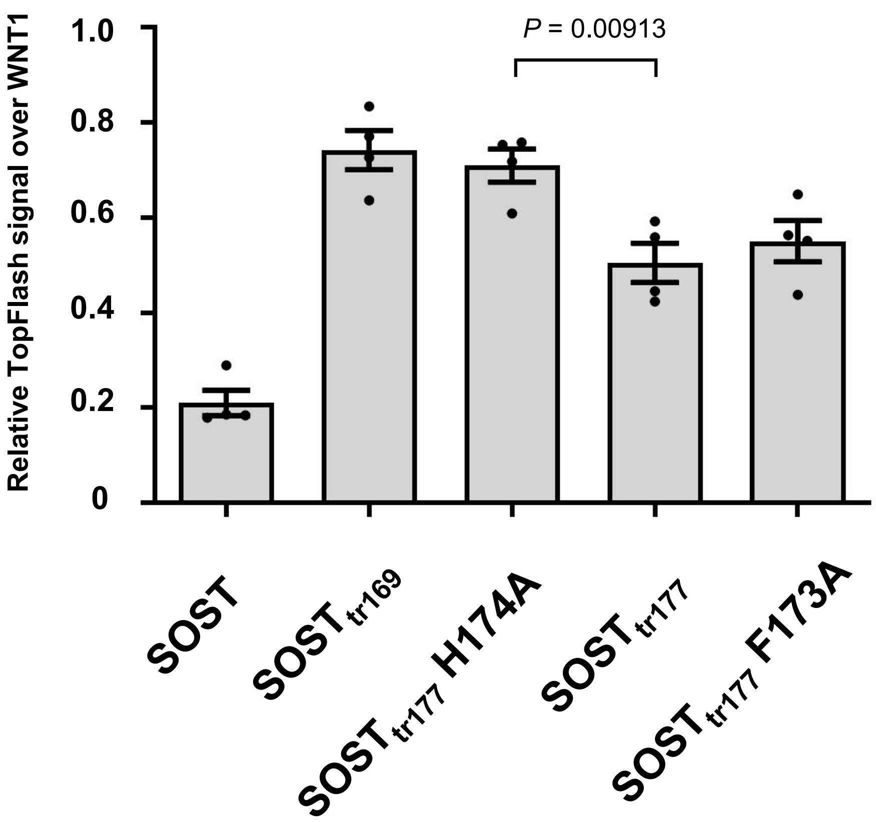

Effect of H174A mutation of $SOST_{tr177}$ on Wnt1-signaling inhibition was detected by TopFlash assays. Each luciferase signal from the co-transfection of Wnt1 and each SOST was normalized to that from the Wnt1 only luciferase signal. Error bars represent SEM from four independent experiments (n=4) and the *p*-value by two-tailed t test is indicated.

Supplementary Figure 16 Expression of LRP6Δacidic mutant in LRP6 knock-out cell

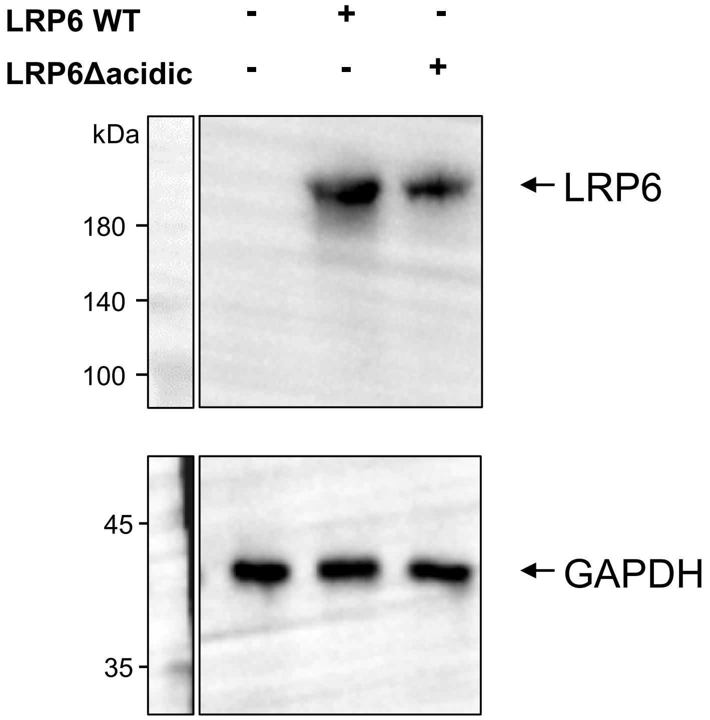

Expression of WT LRP6 and LRP6Δacidic mutant in LRP6 knock-out HEK293T cells was detected by western blot. Molecular weight markers in kDa are shown on the left. Western blots are representative of two independent experiments.

Supplementary Figure 17 Sequence alignment of human LRP5/6 and Xenopus LRP6.

```
hLRP6    20  APLLLYANRRDLRLVDATNGKE-NATIVVGGLEDAAAVDFVFSHGLIYWSDVSEEAIKRTEFNKT-ESVQNVVVSGLLSPDGLACDWLGEKLYWTDSETNRIEVSNL
hLRP5    32  SPLLLFANRRDVRLVDAGGVKL-ESTIVVSGLEDAAAVDFQFSKGAVYWTDVSEEAIKQTYLNQTGAAVQNVVISGLVSPDGLACDWVGKKLYWTDSETNRIEVANL
xLRP6    20  SPLLLYANRRDLRLVDTAGMKG-NSTVVVSGLEDAAAVDFVFSRGLIYWSDVSEEAIKRIDFNKT-GSSQDVVIGGLVSPDGLACDWLGEKLYWTDSETNRIEVSNL

hLRP6   125  DGSLRKVLFWQELDQPRAIALDPSSGFMYWTDWGEVPKIERAGMDGSSRFIIINSEIYWPNGLTLDYEEQKLYWADAKLNFIHKSNLDGTNRQAVVKGSLPHPFALT
hLRP5   135  NGTSRKVLFWQDLDQPRAIALDPAHGYMYWTDWGETPRIERAGMDGSTRKIIVDSDIYWPNGLTIDLEEQKLYWADAKLSFIHRANLDGSFRQKVVEGSLTHPFALT
xLRP6   125  DGSLRKVIFWQELDQPRAIALDPTRGYMYWTDWGEVPKIERAGMDGSVRSVIVDSDIYWPNGLTLDYDEQKLYWADAKLSFIHKANMDGSHRQTVIKGSLAHPFALT
                                                                                         EGF-like Domain
hLRP6   232  LFEDILYWTDWSTHSILACNKYTGEGLREIHSDIFSPMDIHAFSQQRQPNATNPCGIDNGGCSHLCLMSPVKPFYQCACPTGVKLLENGKTCKDGATELLLLARRTD
hLRP5   245  LSGDTLYWTDWQTRSIHACNKRTGGKRKEILSALYSPMDIQVLSQERQPFFHTRCEEDNGGCSHLCLLSPSEPFYTCACPTGVQLQDNGRTCKAGAEEVLLLARRTD
xLRP6   232  LFGDTIYWTDWTTRSILACNKYTGEDRREVDTDIFSPMDIHVFSHLRQPNATNPCAAHNGGCSHLCLMSPMEPFYQCACPTGVRLMEDGKKCMHGATELLLLVRRTD

hLRP6   339  LRRISLDTPDFTDIVLQLED-IRHAIAIDYDPVEGYIYWTDDEVRAIRRSFIDGSGSQFVVTAQIAHPDGIAVDWVARNLYWTDTGTDRIEVTRLNGTMRKILISED
hLRP5   352  LRRISLDTPDFTDIVLQVDD-IRHAIAIDYDPLEGYVYWTDDEVRAIRRAYLDGSGAQTLVNTEINDPDGIAVDWVARNLYWTDTGTDRIEVTRLNGTSRKILVSED
xLRP6   232  LRRISLDTPDFTDIVLPLDD-IRHTIAIDYDPVEGYIYWTDDEVRAIRRTQIDGSSSQYIVTSQVAHPDGIAVDWVARNLYWTDTGTDRIEVTRLNGTMRKILISED
                                                                                                         ↓↓
hLRP6   445  LEEPRAIVLDPMVGYMYWTDWGEIPKIERAALDGSDRVVLVNTSLGWPNGLALDYDEGKIYWGDAKTDKIEVMNTDGTGRRVLVEDKIPHIFGFTLLGDYVYWTDWQ
hLRP5   458  LDEPRAIALHPVMGLMYWTDWGENPKIECANLDGQERRVLVNASLGWPNGLALDLQEGKLYWGDAKTDKIEVINVDGTKRRTLLEDKLPHIFGFTLLGDFIYWTDWQ
xLRP6   445  LDEPRAIVLDPINGYMYWTDWGEIPKIECAAMDGSDRIILVNTSLGWPNGLALDYTEGKIYWGDAKTDKIEVMAIDGTGRRVLVEDKLPHIFGFTLLGDYVYWTDWQ
                            ↓    ↓     ↓                 EGF-like Domain
hLRP6   552  RRSIERVHKRSAE-REVIIDQLPDLMGLKATNVHRVIGSNPCAEENGGCSHLCLYRPQGLRCACPIGFELISDMKTCIVPEA
hLRP5   565  RRSIERVHKVKAS-RDVIIDQLPDLMGLKAVNVAKVVGTNPCADRNGGCSHLCFFTPHATRCGCPIGLELLSDMKTCIVPEA    SOST Loop 2 interaction
xLRP6   552  RRSIERVHKRTGE-REVIIDQLPDLMGLKATNIHKLSGTNGCAENNGGCSHLCLYRPQGPRCACPIGLELINDMKTCIVPEA    SOST C-tail interaction
```

Sequence alignment of LRP5/6 E1E2 domains from human and Xenopus was performed by CLUSTALW program[35]. EGF-like domains are shown as boxed regions and the conserved YWTD motifs in the LRP family are colored red. Residues in LRP6 E1 and E2 interacting with SOST loop 2 and C-tail HNQS are colored green and cyan, respectively. Five mutated acidic residues in the LRP6Δacidic mutant are marked with black arrows.

Supplementary Figure 18 Effect of the WISE C-tail on Wnt1-signaling inhibition.

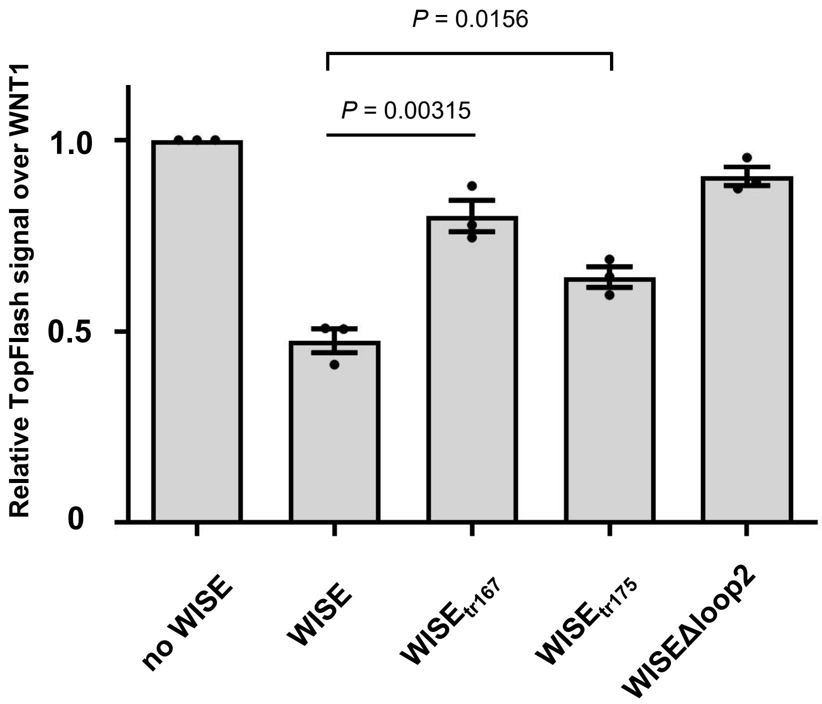

The inhibitory effect of WISE (WT and mutants) on Wnt1 signaling was detected by TopFlash assays. Each luciferase signal from the co-transfection of Wnt1 and each WISE was normalized to that from the Wnt1 only luciferase signal. Error bars represent SEM from three independent experiments (n=3) and the *p*-values by two-tailed t test are indicated.

Supplementary Figure 19 Does-dependent effect of $SOST_{tr169}$ on the 2nd axis formation of *Xenopus* embryo.

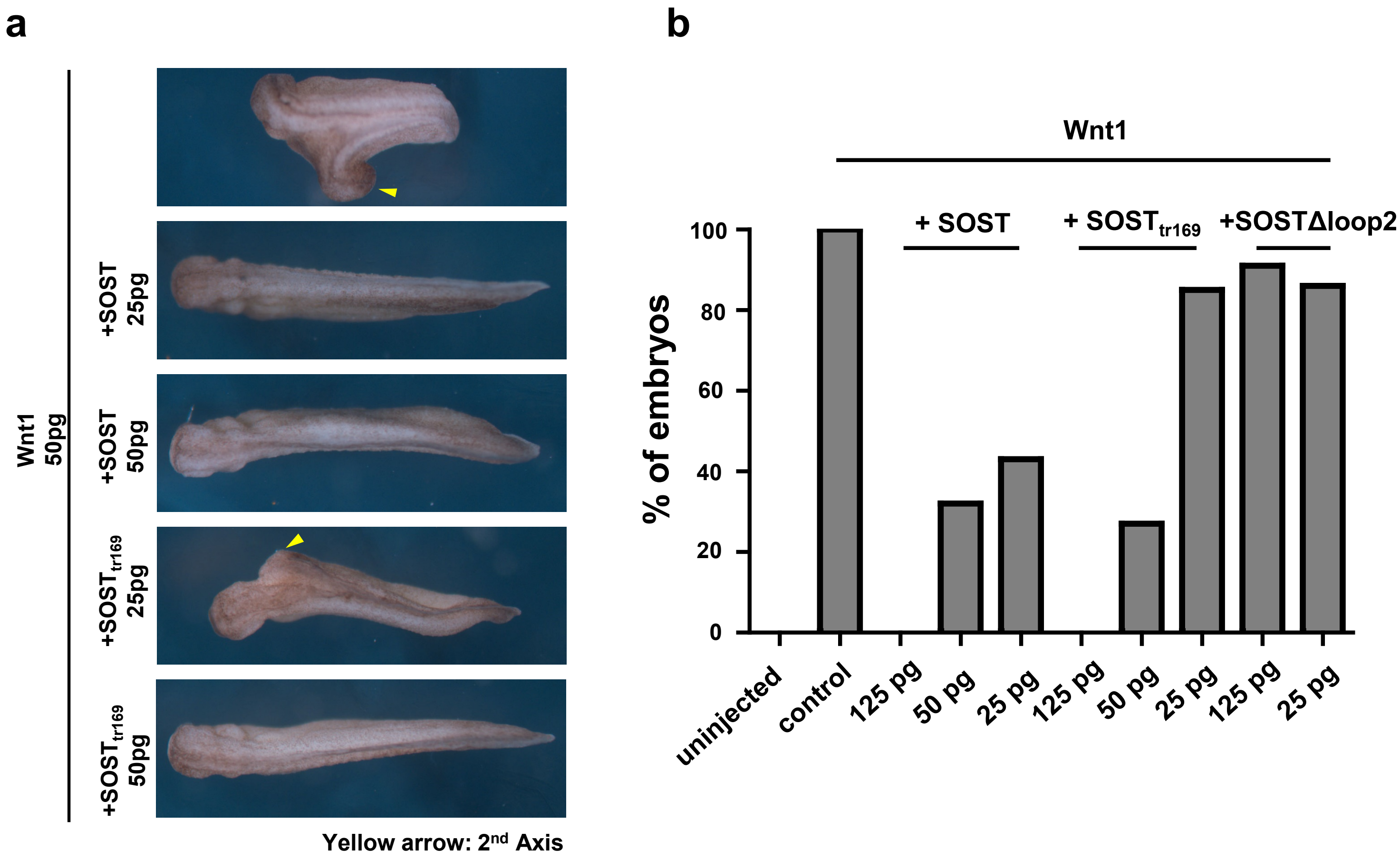

(a) Representative images of *Xenopus* embryos with injections of 50 pg Wnt1 mRNA and two different amounts of WT SOST mRNA or $SOST_{tr169}$ mRNA are shown on the left. Yellow arrow indicates the 2nd axis in *Xenopus*. (b) The percentage of the embryos with second axis are plotted as a bar graph. Each experiment was carried out as follows: uninjected (n = 50), control (n = 18), SOST 125 pg (n = 48), SOST 50 pg (n = 19), SOST 25 pg (n = 51), $SOST_{tr169}$ 125 pg (n = 42), $SOST_{tr169}$ 50 pg (n = 15), $SOST_{tr169}$ 25 pg (n = 53), SOSTΔloop2 125 pg (n = 43), SOSTΔloop2 25 pg (n = 28). ('n' represents the number of embryos used in each experiment.)

Supplementary Figure 20 Inhibition of Wnt3a signaling by SOST at low level of Wnt3a.

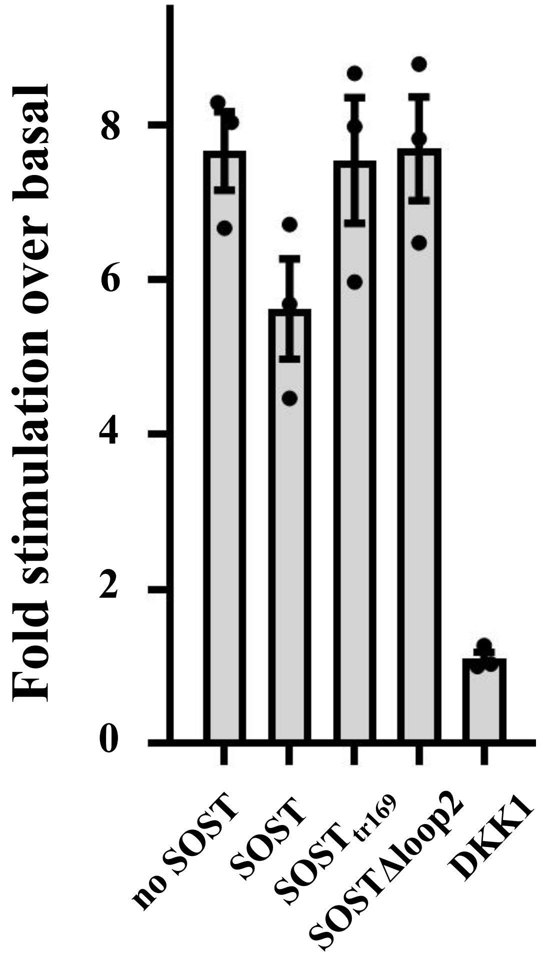

Inhibition of Wnt3a signaling by SOST WT was observed (~25% reduced activity) when low amount of Wnt3a plasmid was transfected. However, Wnt3a-signal inhibition by SOST WT was much lower than that by DKK1. $SOST_{tr169}$ and SOSTΔloop2 did not affect Wnt3a signaling. Data from three independent experiments (n=3) were analyzed and expressed as mean ± SEM.

Supplementary Figure 21 Sequence alignment of four propeller domains of human LRP6

```
E1     20   APLLLYANRRDLRLVDATN-GKENATIVVGGLEDAAAVDFVFSHGLIYWSDVSEEAIKRT
E2    328   TELLLLARRTDLRRISLDTPDFTDIVLQLEDIRHAIAIDYDPVEGYIYWTDDEVRAIRRS
E3    631   EAFLLFSRRADIRRISLET-NNNNVAIPLTGVKEASALDFDVTDNRIYWTDISLKTISRA
E4    933   TTFLLFSQKSAINRMVIDEQQSPDIILPIHSLRNVRAIDYDPLDKQLYWIDSRQNMIRKA

E1     79   EFNKTESVQNVVVSGLLS-----PDGLACDWLGEKLYWTDSETNRIEVSNLDGSLRKVLF
E2    388   FIDG-SGSQFVVTAQIAH-----PDGIAVDWVARNLYWTDTGTDRIEVTRLNGTMRKILI
E3    690   FMNG-SALEHVVEFGLDY-----PEGMAVDWLGKNLYWADTGTNRIEVSKLDGQHRQVLV
E4    993   QEDGSQGFTVVVSSVPSQNLEIQPYDLSIDIYSRYIYWTCEATNVINVTRLDGRSVGVVL

E1    134   WQELDQPRAIALDPSSGFMYWTDWGEV-PKIERAGMDGSSRFIIINSEIYWPNGLTLDYE
E2    442   SEDLEEPRAIVLDPMVGYMYWTDWGEI-PKIERAALDGSDRV-LVNTSLGWPNGLALDYD
E3    744   WKDLDSPRALALDPAEGFMYWTEWGGK-PKIDRAAMDGSERTTLVP-NVGRANGLTIDYA
E4   1053   KGEQDRPRAVVVNPEKGYMYFTNLQERSPKIERAALDGTEREVLFFSGLSKPIALALDSR

E1    193   EQKLYWADAKLNFIHKSNLDGTNRQAVVKGSLPHPFALTLFEDILYWTDWSTHSILACNK
E2    501   EGKIYWGDAKTDKIEVMNTDGTGRRVLVEDKIPHIFGFTLLGDYVYWTDWQRRSIERVHK
E3    802   KRRLYWTDLDTNLIESSNMLGLNREVIAD-DLPHPFGLTQYQDYIYWTDWSRRSIERANK
E4   1113   LGKLFWADSDLRRIESSDLSGANRIVLEDSNILQPVGLTVFENWLYWIDKQQQMIEKIDM
                                                        EGF-like Domain
E1    253   YTGEGLREIHSDIFSPMDIHAFSQ-QRQPN-TNPCGIDNGGCSHLCLMSPVKPFYQCACP
E2    561   RSAEREVIIDQLPDL-MG-LKATN-VHRVIGSNPCAEENGGCSHLCLYRPQG--LRCACP
E3    861   TSGQNRTIIQGHLDYVMDILVFHS-SRQ-SGWNECASSNGHCSHLCLAVPVG-GFVCGCP
E4   1173   TGREGRTKVQARIAQLSDIHAVKELNLQEYRQHPCAQDNGGCSHICLVKGDG-TTRCSCP

E1    312   TGVKLLENGKTCKDGA
E2    616   IGFELISDMKTCI-VP
E3    918   AHYSLNADNRTCS-AP
E4   1232   MHLVLLQDELSCG-EP
```

SOST Loop 2 interaction
SOST C-tail interaction

Sequence alignment of four propeller domains of human LRP6 is presented. EGF-like domains are shown as boxed regions and the conserved YWTD motifs are colored red. Residues interacting with the SOST loop 2 and the C-tail HNQS regions are colored green and cyan, respectively. K684 and L810 in LRP6 E3, and the corresponding residues E73 and A201 in LRP6 E1, respectively are shown in black bold.

Supplementary Figure 22 Structural alignment of SOST-bound LRP6 E1 with LRP6 E3

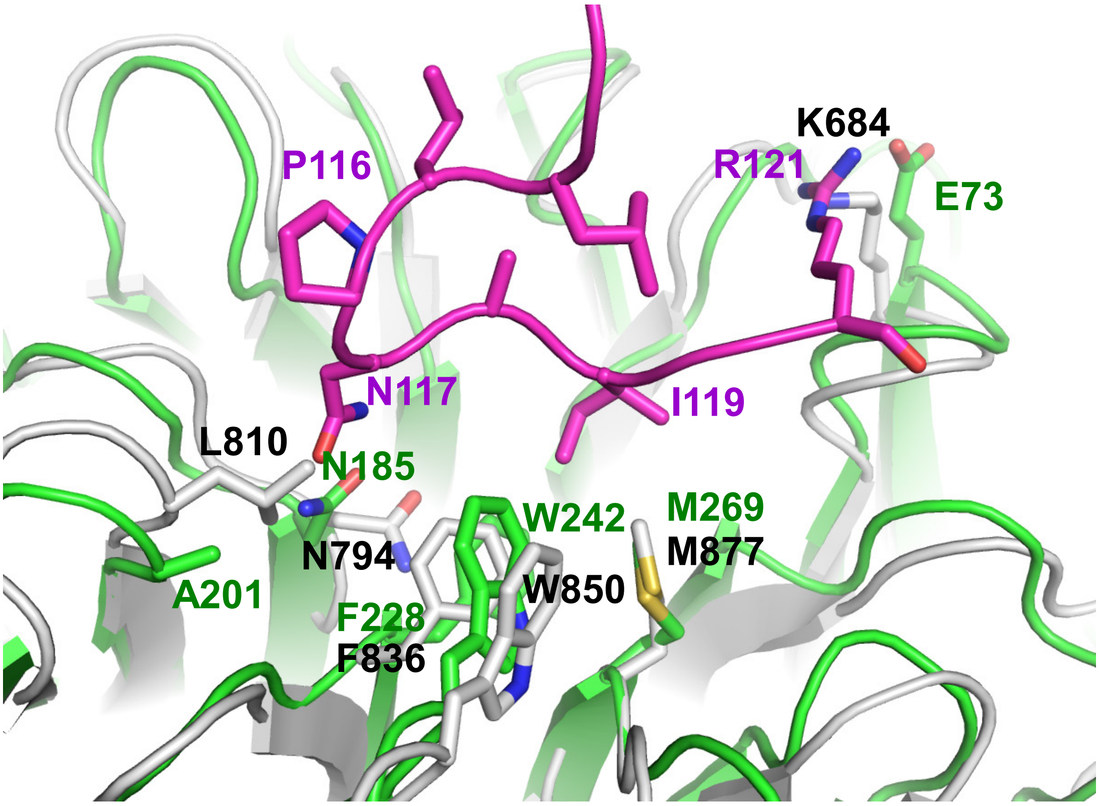

Alignment of LRP6 E3 (grey, PDB ID 3S8Z) into LRP6 E1 (green) of our complex structure (SOST loop 2 in magenta) is presented. LRP6 E1 residues that interact with SOST N117, I119 and R121 and their corresponding residues in LRP6 E3 are shown in sticks. It is unlikely that the similar handshake interaction between N117 of SOST and N185 of LRP6 E1 is formed in LRP6 E3 because nearby bulky L810 collides with N117 of SOST. Also, LRP6 E3 does not have the appropriate residue corresponding to E73 in E1, which makes an ionic interaction with R121 of SOST, and instead has K684.

Supplementary Figure 23 B-factor putty representation of the complex

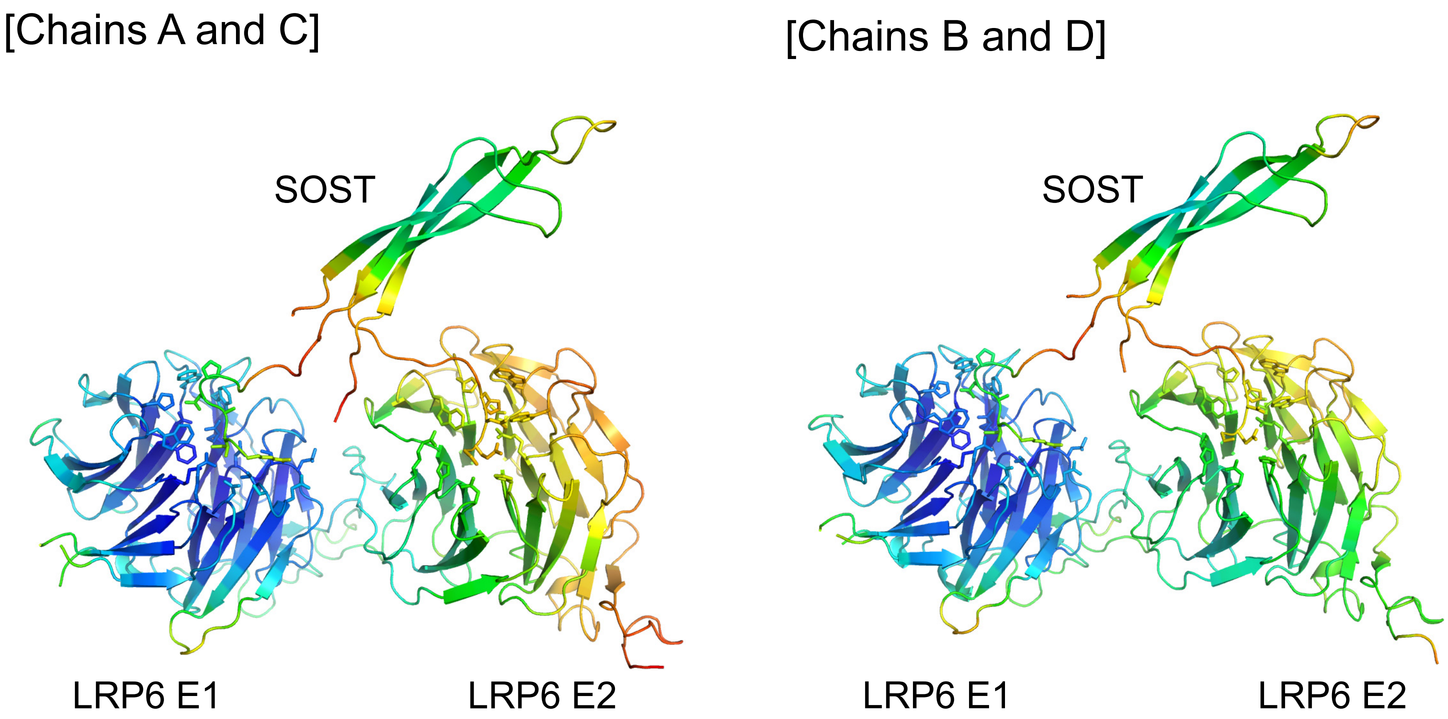

The structure of two complex molecules in an asymmetric unit is drawn in cartoon putty representations; blue represents the lowest and red the highest B-factor value.

Supplementary Table 1. Construct information

Construct Name	Residue Number
SOST	Q24-Y213
$SOST_{tr204}$	Q24-A204
$SOST_{tr187}$	Q24-A187
$SOST_{tr178}$	Q24-E178
$SOST_{tr177}$	Q24-S177
$SOST_{tr169}$	Q24-R169
SOSTΔloop2	Q24-Y213 (substitution of R113-P127 with GSGG)
SOSTΔHNQS	Q24-Y213 (substitution of R172-E178 with GSGG)
SOSTΔbasic	Q24-Y213 (K180A, R188A, K191A, R193A, K194A, R196A, R198A, R200A, K203A)
$SOST_{tr177}$ F173A	Q24-S177 (F173A)
$SOST_{tr177}$ H174A	Q24-S177 (H174A)
$SOST_{tr177}$ N175K	Q24-S177 (N175K)
WISE	F24-S206
$WISE_{tr175}$	F24-S175
$WISE_{tr167}$	F24-R167
WISEΔloop2	F24-S206 (substitution of L110-E130 with GSGG)
LRP6Δacidic	A20-A1370 (E529K, D530K, E564K, E566K, D570K)
LRP6 E1E2	P21-P630
LRP6 E1E2 H404K	P21-P630 (H404K)
LRP6 E1	P21-D325

Supplementary Table 2. Dissociation constant of each SOST protein for each LRP6 measured by MST.

**Construct**	**LRP6 E1E2 (μM)**	**LRP6 E1 (μM)**
Smo-SOST	0.17 ± 0.05	0.78 ± 0.38
Smo-SOST$_{tr169}$	0.90 ± 0.29	0.87 ± 0.16
Smo-SOST$_{tr177}$	0.68 ± 0.17	
Smo-SOST$_{tr178}$	0.68 ± 0.16	
Smo-SOST$_{tr187}$	0.60 ± 0.17	
Smo-SOST$_{tr204}$	0.29 ± 0.07	
Smo-SOSTΔloop2	1.0 ± 0.1	
Smo	N.B.	
MBP-SOST	0.17 ± 0.08	
MBP	N.B.	
Circularized loop2 peptide	5.5 ± 1.1	5.2 ± 1.3
C-tail peptide	5.8 ± 1.3	N.B.
Histone peptide	N.B.	

N.B. represents “No apparent binding”

Supplementary Table 3. List of primer sequences for cloning

Name	Forward primer (5'-3')*	Reverse primer (5'-3')*	Purpose
**PCR primers for subcloning**			
LRP6 E1E2	CCGAGGATCCTTTGTTGCTTTATGCAAACAGA	GGTTGAATTCTTAATGGTGATGGTGGTGGTGGTGATGGTGTGGGACAATGCAGGTCTTCATG	Crystallization
$SOST_{tr177}$	TTAATCTAGACAGGGGTGGCAGGCG	AATTGCGGCCGCTTACGACTGGTTGTGGAAGCGG	Crystallization
LRP6 E1	CCGAGGATCCTTTGTTGCTTTATGCAAACAGA	GGCCGAATTCCTAATGGTGATGGTGGTGGTGATCTTTGCAGGTTTTTCCATTCTC	Expression
SOST	GGTTGAATTCTTCAGGGGTGGCAGGCG	TTAAGCGGCCGCCTAGTAGGCGTTCTCCAGCTCGG	Expression
$SOST_{tr204}$	GGTTGAATTCTTCAGGGGTGGCAGGCG	TTAAGCGGCCGCTTAGGCTTTGGCGCTCCGGGCGCG	Expression
$SOST_{tr187}$	GGTTGAATTCTTCAGGGGTGGCAGGCG	TTAAGCGGCCGCTTAAGCGGCCTCGGTCCCGAA	Expression
$SOST_{tr178}$	GGTTGAATTCTTCAGGGGTGGCAGGCG	TTAAGCGGCCGCTTACTCCGACTGGTTGTGGAAG	Expression
$SOST_{tr177}$	GGTTGAATTCTTCAGGGGTGGCAGGCG	TTAAGCGGCCGCTTACGACTGGTTGTGGAAGCGG	Expression
$SOST_{tr169}$	GGTTGAATTCTTCAGGGGTGGCAGGCG	TTAAGCGGCCGCTTAGCGCTTGCACTTGCACGAG	Expression
SOST C-tail	TTAAGAATTCTCACCCGCTTCCACAACCAGT	ATGTAAGCTTCTAGTAGGCGTTCTCCAGCTCGG	Expression
SOST N175K	GGTTGAATTCTTCAGGGGTGGCAGGCG	GGTTGCGGCCGCATTAGTGGTGATGGTGATGATGGGATTGTTTGTGGAAGCGGGTGAG	Expression
SOST	TTAAGAATTCTATGCAGCTCCCACTGGCCC	ATGTAAGCTTCTAGTAGGCGTTCTCCAGCTCGG	TopFlash
$SOST_{tr204}$	TTAAGAATTCTATGCAGCTCCCACTGGCCC	ATGTAAGCTTTAGGGCTTTGGCGCTCCGGGCGCG	TopFlash
$SOST_{tr187}$	TTAAGAATTCTATGCAGCTCCCACTGGCCC	ATGTAAGCTTTAGAGCGGCCTCGGTCCCGAA	TopFlash
$SOST_{tr178}$	TTAAGAATTCTATGCAGCTCCCACTGGCCC	ATGTAAGCTTTAGCTCCGACTGGTTGTGGAAG	TopFlash
$SOST_{tr177}$	TTAAGAATTCTATGCAGCTCCCACTGGCCC	ATGTAAGCTTTAGCGACTGGTTGTGGAAGCGG	TopFlash
$SOST_{tr169}$	TTAAGAATTCTATGCAGCTCCCACTGGCCC	ATGTAAGCTTTAGGCGCTTGCACTTGCACGAG	TopFlash
WISE	GCCTGAATTCTATGCTTCCTCCTGCCA	GGCCAAGCTTCTAACTCATGCTGTGCTTG	TopFlash
WISEtr175	GCCTGAATTCTATGCTTCCTCCTGCCA	GGCTAAGCTTTTAGGACTCGTTGTGCTGC	TopFlash
WISEtr167	GCCTGAATTCTATGCTTCCTCCTGCCA	GGCCAAGCTTTTACCTCTTGCACTTGCAG	TopFlash
**Secondary PCR primers for deletion and point mutation**			
SOSTΔloop2	CCCACTACCACCTGAACCCGCCGGGCCGCACTGGC	CCGGCGGGTTCAGGTGGTAGTGGGCCCGACTTCCGCTG	Expression/ TopFlash
SOSTΔHNQS	GGTGGTCTCAAGGACTTCGGGACC	TGAACCGGTGAGGCGCTTGCACTT	TopFlash
WISEΔloop2	CCAGTGGGTTCAGGTGGTTGGCGGTGTGTCAATGA	CCGCCAACCACCTGAACCCACTGGCAGGGGCAAGC	TopFlash
SOST N117A	GCTTCCACGCCCAGTCGGAGCTCAAGGACTT	CCGACTGGGCGTGGAAGCGGGTGAGG	TopFlash
LRP6 H404K	ATTGCCAAACCTGATGGTATTGCTGT	CCATCAGGTTTGGCAATTTGAGCAGTGA	Expression
**PCR primers for site directed mutagenesis**			
SOST F173A	CCTCACCCGCGCCCACAACCAGTC	CGCTTGCACTTGCACGAG	TopFlash
SOST H174A	CACCCGCTTCGCCAACCAGTCGGAG	AGGCGCTTGCACTTGCAC	TopFlash

*Restriction sites are underlined

Supplementary Table 4. Data collection and refinement statistics

Data collection	LRP6 E1E2-SOST$_{tr177}$
Wavelength (Å)	1.033
Space group	P64
Unit cell parameters	
a, b, c (Å)	252.8, 252.8, 86.3
Resolution (Å) (last shell)	30-3.8 (3.94-3.8)
Unique reflections	31247 (3106)
Completeness (%)	99.6 (99.4)
Multiplicity	4.3 (4.2)
I/s(I)	6.5 (2.0)
R_{merge}[a]	0.12 (0.42)
$CC_{1/2}$	0.98 (0.78)
Wilson B-factor (Å^2)	87
Refinement	
No. of reflections working set (test set)	31258 (2465)
R_{cryst}/ R_{free}[b]	0.21/0.26
Bond length rmsd from ideal (Å)	0.003
Bond angle rmsd from ideal (°)	0.73
Average B-factors (Å^2), LRP6	97 (chain A), 90 (chain B)
SOST	117 (chain C), 110 (chain D)
Ramachandran analysis[c]	
% favored regions	86.5
% allowed regions	13.4
% outliers	0.1

rmsd, root-mean square deviation.

[a] $R_{merge} = \sum_h \sum_i | I_i(h) - \langle I(h) \rangle | / \sum_h \sum_i I_i(h)$, where $I_i(h)$ is the *i*th measurement of reflection *h*, and $\langle I(h) \rangle$ is the weighted mean of all measurements of *h*.

[b] $R = \sum_h |F_{obs}(h)| - |F_{calc}(h)| | / \sum_h |F_{obs}(h)|$. R_{cryst} and R_{free} were calculated using the working and test reflection sets, respectively.

[c] As defined in MolProbity[6]

Supplementary References

1. Matthews, B.W. Solvent content of protein crystals. J Mol Biol **33**, 491-7 (1968).
2. Thompson, J.D., Higgins, D.G. & Gibson, T.J. CLUSTAL W: improving the sensitivity of progressive multiple sequence alignment through sequence weighting, position-specific gap penalties and weight matrix choice. *Nucleic Acids Res* **22**, 4673-80 (1994).
3. Baker, N.A., Sept, D., Joseph, S., Holst, M.J. & McCammon, J.A. Electrostatics of nanosystems: application to microtubules and the ribosome. *Proc Natl Acad Sci U S A* **98**, 10037-41 (2001).
4. Pettersen, E.F. et al. UCSF Chimera--a visualization system for exploratory research and analysis. *J Comput Chem* **25**, 1605-12 (2004).
5. DeLano, W.L. The PyMOL Molecular Graphics System. DeLano Scientific (San Carlos, CA. USA). (2002).
6. Williams, C.J. et al. MolProbity: More and better reference data for improved all-atom structure validation. *Protein Sci* **27**, 293-315 (2018).
