# Supplementary material for: Sclerostin inhibits Wnt signaling through tandem interaction with two LRP6 ectodomains
Source: Nat Commun. 2020 Oct 23;11:5357. doi: 10.1038/s41467-020-19155-4 (PMC7585440; doi:10.1038/s41467-020-19155-4)
Supplement: Supplementary file 2 — Reporting Summary [file 41467_2020_19155_MOESM2_ESM.pdf]

nature research

Corresponding author(s): Hee-Jung Choi

Last updated by author(s): Sep 18, 2020

Reporting Summary

Nature Research wishes to improve the reproducibility of the work that we publish. This form provides structure for consistency and transparency in reporting. For further information on Nature Research policies, see our Editorial Policies and the Editorial Policy Checklist.

Statistics

For all statistical analyses, confirm that the following items are present in the figure legend, table legend, main text, or Methods section.

n/a	Confirmed	
☐	☒	The exact sample size (n) for each experimental group/condition, given as a discrete number and unit of measurement
☐	☒	A statement on whether measurements were taken from distinct samples or whether the same sample was measured repeatedly
☐	☒	The statistical test(s) used AND whether they are one- or two-sided<br>*Only common tests should be described solely by name; describe more complex techniques in the Methods section.*
☒	☐	A description of all covariates tested
☐	☒	A description of any assumptions or corrections, such as tests of normality and adjustment for multiple comparisons
☐	☒	A full description of the statistical parameters including central tendency (e.g. means) or other basic estimates (e.g. regression coefficient) AND variation (e.g. standard deviation) or associated estimates of uncertainty (e.g. confidence intervals)
☐	☒	For null hypothesis testing, the test statistic (e.g. F, t, r) with confidence intervals, effect sizes, degrees of freedom and P value noted<br>*Give P values as exact values whenever suitable.*
☒	☐	For Bayesian analysis, information on the choice of priors and Markov chain Monte Carlo settings
☒	☐	For hierarchical and complex designs, identification of the appropriate level for tests and full reporting of outcomes
☒	☐	Estimates of effect sizes (e.g. Cohen's d, Pearson's r), indicating how they were calculated

Our web collection on statistics for biologists contains articles on many of the points above.

Software and code

Policy information about availability of computer code

Data collection	X-ray diffraction data were collected at the Pohang Accelerator Laboratory (PAL, South Korea) and the Advanced Photon Source (APS, USA). The data set was indexed, integrated, scaled and merged using XDS. Data collection statistics are shown in Supplementary Table 4. Crosslinking mass analysis was performed with Orbitrap Fusion Lumos mass spectrometer (Thermo Scientific) coupled with NanoAcquity UPLC system (Waters, Milford).
Data analysis	XDS (version march 15, 2019), Aimless (version 7.1.003), Phaser (version 1.16), Coot (version 0.9), Phenix (version 1.16), GraphPadPrism (version 5.0), ASTRA (version 6), GalaxyLoop, PIPER-FlexPepDock (version 2016.20.58704), GalaxyPepDock-ab-initio, GalaxyRefineComplex, msConvert (version 3.0.19241-cce51a42d), MS-GF+ (version 2017.01.27), Xcalibur Qual Browser (version 4.1.50).

For manuscripts utilizing custom algorithms or software that are central to the research but not yet described in published literature, software must be made available to editors and reviewers. We strongly encourage code deposition in a community repository (e.g. GitHub). See the Nature Research guidelines for submitting code & software for further information.

Data

Policy information about availability of data

All manuscripts must include a data availability statement. This statement should provide the following information, where applicable:

- Accession codes, unique identifiers, or web links for publicly available datasets
- A list of figures that have associated raw data
- A description of any restrictions on data availability

Coordinate file and structure factor file have been deposited in the Protein Data Bank under accession number of 6L6R. They will be available upon publication. Source data are provided as a Source Data file. Other data supporting the findings of this work are available from the corresponding author upon reasonable request.

Field-specific reporting

Please select the one below that is the best fit for your research. If you are not sure, read the appropriate sections before making your selection.

- [x] Life sciences
- [ ] Behavioural & social sciences
- [ ] Ecological, evolutionary & environmental sciences

For a reference copy of the document with all sections, see nature.com/documents/nr-reporting-summary-flat.pdf

Life sciences study design

All studies must disclose on these points even when the disclosure is negative.

Sample size	In figures 5a and 5b, each sample contains 15-53 of embryos at the four-cell stage for axis duplication assay. More details on sample size used in these experiments are described in Figure 5a and Supplementary Figure 19 legends. In figures 5c and 5d, 20 animal caps were used for qPCR analysis. For the experiments with Xenopus embryos, no statistical methods were used to predetermined sample size. Sample size was chosen by following the literature in the field.
Data exclusions	No data was excluded from the analysis
Replication	Three replicates were performed for each affinity measurement and each luciferase assay. Axis duplication assay and qPCR analysis were each independently performed three times. We confirmed that similar results were obtained from these repeated experiments.
Randomization	Xenopus embryos were randomly selected for microinjection
Blinding	In this study, all experiments using Xenopus embryo are not influenced by the tester’s expectation.

Reporting for specific materials, systems and methods

We require information from authors about some types of materials, experimental systems and methods used in many studies. Here, indicate whether each material, system or method listed is relevant to your study. If you are not sure if a list item applies to your research, read the appropriate section before selecting a response.

Materials & experimental systems

n/a	Involved in the study	
[ ]	[x]	Antibodies
[ ]	[x]	Eukaryotic cell lines
[x]	[ ]	Palaeontology and archaeology
[ ]	[x]	Animals and other organisms
[x]	[ ]	Human research participants
[x]	[ ]	Clinical data
[x]	[ ]	Dual use research of concern

Methods

n/a	Involved in the study	
[x]	[ ]	ChIP-seq
[x]	[ ]	Flow cytometry
[x]	[ ]	MRI-based neuroimaging

Antibodies

Antibodies used	anti-GAPDH Antibody (sc-47724, Santa Cruz Biotechnology), anti-His-Tag (27E8) Mouse mAb (2366, Cell Signaling), anti-LRP6 (C47E12) Rabbit mAb (3395, Cell Signaling), anti-Sclerostin Monoclonal Antibody (220902, Invitrogen)
Validation	All antibodies were validated by purified target proteins in western blots.

Eukaryotic cell lines

Policy information about cell lines

Cell line source(s)	Human Embryonic Kidney (HEK) 293A cell line from Invitrogen was used for cell-based assays. LRP6 knock-out cell line was generated from HEK 293T cell line (ATCC).
Authentication	HEK 293A (Invitrogen) and HEK 293T (ATCC) cell lines were authenticated by STR method. Generated LRP6 knock-out HEK293T cell line in this study was verified by western blot.
Mycoplasma contamination	LRP6 knock-out HEK 293T cell line was not tested for mycoplasma contamination. HEK293A cell line was mycoplasma negative.
Commonly misidentified lines (See ICLAC register)	No commonly misidentified cell lines were used in the study.

Animals and other organisms

Policy information about studies involving animals; ARRIVE guidelines recommended for reporting animal research

Laboratory animals	Xenopus laevis (males and females), mature (from 1 to 4 years old), from NASCO
Wild animals	Wild animals were not used
Field-collected samples	Field-collected samples were not used
Ethics oversight	Xenopus laevis was used following the instruction from the POSTECH IACUC (Institutional Animal Care and Use Committees) (Korea) after achieving the certification for ethical handling.

Note that full information on the approval of the study protocol must also be provided in the manuscript.
